# Profiling of Integrin Isoforms on Lung‐Tropic Exosomes by Spectrally and Kinetically Multiplexed Single‐Molecule Imaging

**DOI:** 10.1002/advs.202515129

**Published:** 2025-12-31

**Authors:** Songlin Liu, Haixin Wang, Qin Shentu, Liang Yuan, Jiangshan Tie, Li Li, Rui Ai, Bochen Ma, Lubin Qi, Yifei Jiang, Xiaohong Fang

**Affiliations:** ^1^ School of Chemistry and Materials University of Science and Technology of China Hefei Anhui 230026 P. R. China; ^2^ Hangzhou Institute of Medicine (HIM) Chinese Academy of Sciences Hangzhou Zhejiang 310022 P. R. China; ^3^ School of Molecular Medicine Hangzhou Institute for Advanced Study UCAS Hangzhou 310024 P. R. China; ^4^ Beijing National Research Center for Molecular Sciences Institute of Chemistry Key Laboratory of Molecular Nanostructure and Nanotechnology Chinese Academy of Science Beijing 100190 P. R. China

**Keywords:** DNA‐PAINT, exosome, integrin, multiplexed detection, single‐molecule imaging

## Abstract

Since the first report that integrins on exosomes can potentially dictate their organ‐tropic transport, there have been great interest in obtaining a better understanding of this phenomenon. However, integrins have many isoforms, which are heterogeneously distributed among individual exosomes with relatively low abundances. As a result, it is difficult to profile their expression landscape at single exosome level and study their relationship with organ specificity. To overcome this limitation, a spectrally and kinetically multiplexed single‐molecule imaging method is developed, which for the first time achieved simultaneously imaging of 12 exosomal proteins at an unparalleled single‐copy resolution, allowing systematic profiling of integrins on individual exosomes. Using this method, integrin expression profile of various types of cellular exosomes are characterized. Using machine learning algorithms, clustering analysis is performed to identify key subpopulations of each type of exosomes. It is found that co‐occurrence integrin α6 and the pairing β counterpart on single exosomes is essential for effective lung targeting. In contrast, exosomes with primary αv dimer or unpaired integrins show almost no lung targeting behavior. Overall, the method allows profiling of integrins on single exosomes with unprecedented resolution. The information gained can facilitate the development of exosome‐based biopsies and therapies.

## Graphical abstract

A spectrally and kinetically multiplexed single‐molecule imaging method is developed, which achieved simultaneous imaging of 12 exosomal proteins at an unparalleled single‐copy resolution. It is found that co‐occurrence of α6 integrin and the pairing β counterpart on single exosomes is essential for lung targeting, providing valuable information for exosome‐based biopsies and therapies.

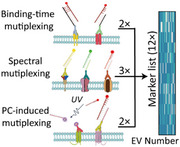

## 1 Introduction

Exosomes, nanovesicles within the 30 to 150 nm size range, play a pivotal role in intercellular communication and influence various biological activities, including signaling, immune responses, and cellular waste disposal.^[^ ^1^, ^2^, ^3^, ^4^ ^]^ One of the most intriguing features of exosomes is their targeted transport between cells and organs.^[^ ^5^ ^]^ Such process, orchestrated by the proteins on the exosome surface, has drawn considerable research interests due to its application potential in drug delivery and cancer metastasis diagnosis.^[^ ^6^, ^7^, ^8^, ^9^ ^]^ Integrins, a family of proteins respond for cell‐cell adhesion, have been shown to exist on exosome surface and play an important role in their organ‐selective uptake.^[^ ^10^, ^11^ ^]^ However, integrins have many isoforms, which are heterogeneously distributed among individual exosomes.^[^ ^12^ ^]^ Profiling of their expression landscape at single exosome level is required in order to elucidate their relationship with the organ‐specific exosome transport.

Analysis of proteins on single exosomes is hindered by their small size and the relatively low protein abundance.^[^ ^13^ ^]^ Many methods have been developed to amplify protein signals on exosomes, which either involve inducing exosome aggregation or employs enzymatic reactions.^[^ ^14^, ^15^, ^16^, ^17^ ^]^ However, since exosomes are highly heterogeneous, signal amplification by aggregation could result in loss of bio‐information. For enzymatic methods, variations in the amplification efficiency could affect the fidelity of the analysis. Compared to the above methods, single‐molecule imaging exhibits very high sensitivity, and can measure heterogeneity within samples, making it uniquely suited for single‐exosome analysis.^[^ ^18^, ^19^, ^20^, ^21^ ^]^ Conventional single‐molecule imaging methods have limited multiplexing capability. Cyclic imaging methods have been developed to increase the number of imaging targets.^[^ ^22^, ^23^ ^]^ These methods, however, have low throughput and prone to colocalization errors induced by repeated staining and washing processes. Overall, there is currently lack of a method that can efficiently profile a large number of proteins on individual exosomes with single‐molecule sensitivity.^[^ ^24^, ^25^ ^]^

To overcome this limitation, we developed a single‐step, highly multiplexed single‐molecule imaging method based on DNA ‐ based point accumulation for imaging in nanoscale topography (DNA‐PAINT). In addition to the conventional 3 color spectra multiplexing, we utilized DNA binding and photo‐cleavage kinetics for resolution of additional markers. Specifically, we conjugated antibodies with DNA strands of different lengths to induce various binding times, and attributed them to different markers. In addition, we conjugated the same DNA strands with different antibodies using non‐cleavable and photo‐cleavable linkers, which allows resolution of markers based on binding frequency change upon photo‐cleavage. Since imaging wavelength, binding time and photo‐cleavage response are orthogonal parameters, our method for the first time achieved simultaneous imaging of up to 12 surface markers on single exosome within a single‐step incubation. Repeated binding of DNA strands also allows ultra‐sensitive detection of protein markers down to a single copy, and digital assessment of protein copy number based on binding event count.^[^ ^26^ ^]^ Compared with commercial single‐vesicle analytical methods, this study offers a dramatically enhanced multiplexing capability, while maintaining high analytical sensitivity, allowing simultaneous quantification of a large number of biomarkers on single exosome (see Table S1, Supporting Information for detailed comparison). Furthermore, our method can be easily integrated with the various spectral‐resolved imaging approaches, such as spectral‐resolved super‐resolution microscopy, to achieve even higher‐order of multiplexing.^[^ ^27^, ^28^, ^29^ ^]^ This would empower the validation and analysis of complex biological mechanisms involving extensive biomarker panels.

Using this method, we characterized integrin expression profile of various types of cellular exosomes that exhibit different lung targeting effect. Among the integrin isoforms, we focused on αv, α6 and 6 types of β isoforms that can form hetero‐dimer within them. αv and α6 integrins are suspected to promote lung metastasis of various types of cancer, though their distribution on individual lung‐tropic exosomes has not been studied.^[^ ^30^, ^31^ ^]^ We found that integrin isoforms are heterogeneously expressed with low copy number on single exosomes. Using machine learning algorithms, clustering analysis was performed to identify key subpopulations of each type of cellular exosomes. We observed that exosomes that contain major subpopulations with paired α6 heterodimer showed clear lung accumulation. In contrast, exosomes with primary αv dimer or unpaired integrins showed almost no lung targeting behavior. While previous researches have linked α6 integrin with lung‐tropism, it is unclear whether they mostly exist as monomers or paired dimers on single exosomes, due to the lack of characterization method. We showed that the lung‐tropism of exosomes is not dictated by α6 integrin alone. Instead, co‐occurrence α6 integrin and the pairing β counterpart on single exosomes is essential for effective lung targeting. Overall, we developed a highly multiplex single‐molecule imaging method and systematically studied integrin expression on various cellular exosomes, which represents the first attempt to understand integrin‐mediated lung targeting at the single exosome level. The information gained could contribute to the development of exosome‐based biopsies and therapies.

## 2 Results and Discussion

### 2.1 Imaging Principle

**Scheme 1:**
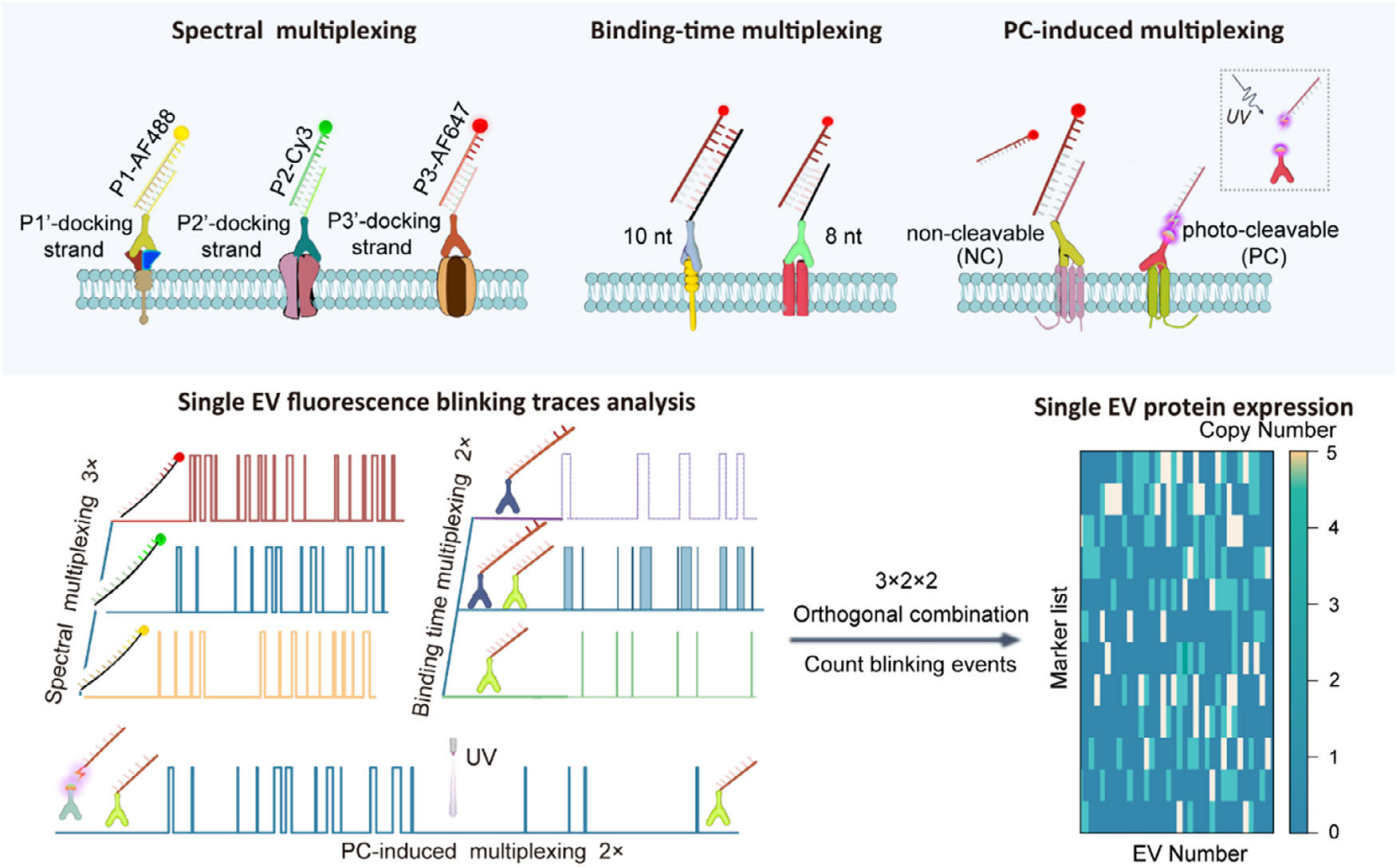
Schematic illustration of distinguishing 12 biomarkers on single exosomes using a DNA‐PAINT method that combines spectral (emission color) and kinetic (binding time and UV‐induced frequency change) parameters.

We developed an advanced DNA‐PAINT‐based single‐molecule imaging method, which utilizes spectral (emission color) and kinetic parameters (binding duration and UV‐induced frequency modulation) to discriminate multiplexed detection of 12 biomarkers on single exosomes (**Scheme** **1**). The methodology builds upon the fundamental principle of DNA‐PAINT, which achieves stochastic blinking of molecular targets through transient hybridization between freely diffusing dye‐labeled imager strands and surface‐immobilized complementary docking strands. First, we used DSPE‐PEG‐Biotin to functionalize exosome surface with biotin, enabling immobilization of exosomes onto a streptavidin‐coated substrate. Subsequently, antibody‐DNA conjugates are used to target different exosomal proteins. Each conjugate comprises two key elements: the type of linker between the antibody and the DNA (non‐cleavable [NC] or photo‐cleavable [PC]), and the number of hybridization base pairs on the DNA strand (8 or 10 nt). During imaging, multicolor dye‐conjugated DNA strands (P1‐Alexa Flour 488 [AF488], P2‐Cy3, P3‐Alexa Flour 647 [AF647]) that are complementary to the DNA strands on the antibodies (docking strands) were added to the solution, which induces dynamic interactions between the imager strands and the targets. Repeated binding of the DNA strands allows ultra‐sensitive detection of protein markers. In addition to the spectral multiplexing, different binding times induced by the various hybridization lengths enable differentiation of additional markers. The non‐cleavable and photo‐cleavable linkers between antibodies and DNAs also allows resolution of two markers based on binding frequency change upon photo‐cleavage. Since imaging wavelength (3), binding time (2) and photo‐cleavage response (2) are orthogonal parameters, our method allows simultaneous imaging of up to 12 (3 × 2 × 2) surface markers on single exosomes within a single‐step incubation. Streptavidin‐conjugated poly(9,9‐dioctylfluorene) (PFO) polymer dots were used for labeling of the exosomal membrane. The PFO polymer dots emit in the 400–460 nm range, which do not interfere with the dyes conjugated to the imager strands (AF488, Cy3, AF647), and are ultra‐photostable, allowing prolonged marking of the exosomes’ positions and convenient drift correction.^[^ ^32^, ^33^ ^]^

In addition to the high sensitivity and multiplexity, another key strength of the method is the ability to quantify protein copy number on each exosome. Basically, without any multiplexing and under the same imaging condition (imager concentration and buffer), the number of binding sites on each exosome is correlated with the frequency of binding events, which can be calculated using the formula *N* = (*k_on_* × *c* × τ_d*_)^−1^.^[^ ^34^ ^]^ The influx rate ξ = *k_on_* × *c*, was calculated for each docking‐imager pair using DNA nanostructures with known binding sites as standards (Methods and Table S2 and Figures S1 and S2, Supporting Information). Then, the average dark‐state time τ_d*_ derived from the fluorescence traces was used to calculate the number of binding sites *N*. When multiple markers are imaged simultaneously, the same principle can be used for calculation, though the procedures are more complicated, which will be discussed in details below.

### 2.2 Spectral Multiplexing

**Figure 1:**
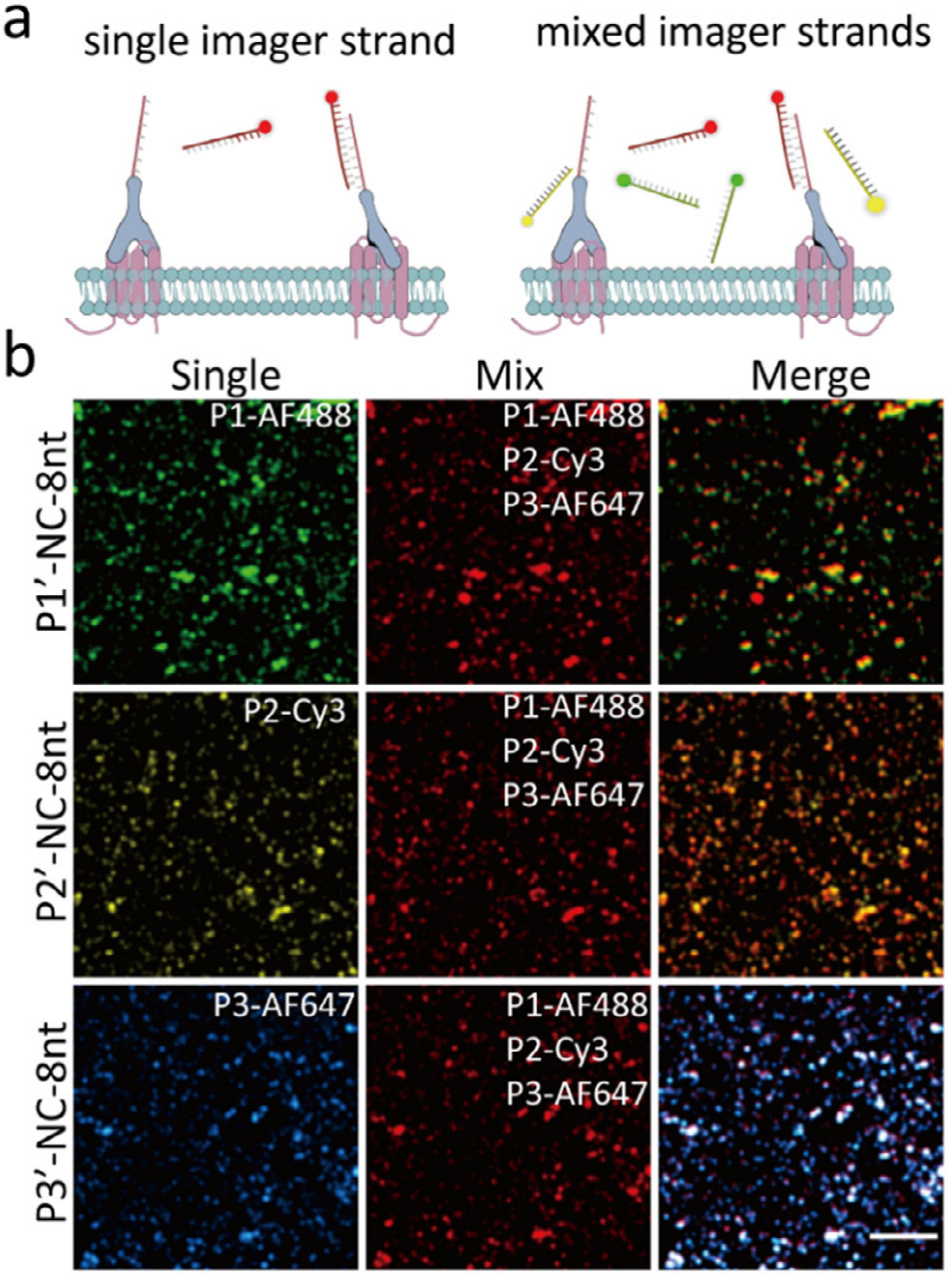
Multiplexed single‐molecule imaging of exosome with mixed imager strands. a) Schematic of DNA‐PAINT imaging of CD9 with single imager strands (left) and a mixed imager cocktail (right). b) Localization maps of CD9 on A549 exosomes using single imager strands and a mixed imager cocktail. The nearly identical localization patterns confirm minimal cross‐interaction and validate the multiplexing capability. Scale bars, 5 µm. Statistical analysis was carried out using Imager J Software.

Conventional DNA‐PAINT uses sequential replacement of imager strands for multiplexed imaging, which is time consuming.^[^ ^35^ ^]^ One of the most straightforward way to achieve multiplexing without sacrificing the imaging efficiency is to use multicolor dye‐conjugated imager strands (**Figure** **1**a). However, since this involves using several different imager strands simultaneously, careful sequence design is required to avoid undesirable interactions between the strands.^[^ ^36^ ^]^ For validation of this approach, we prepared three types of CD9 antibody‐DNA conjugates, each contains a distinct docking strands (P1'‐NC‐8 nt, P2'‐NC‐8 nt and P3'‐NC‐8 nt), and used them to label A549 cell exosomes. Imager strands that are complementary to the different docking strands were conjugated with different color of dyes (P1‐AF488, P2‐Cy3 and P3‐AF647). First, the imaging laser intensity was optimized to ensure no spectral cross‐talk between different fluorescent channels for each imager strand (Figure S3, Supporting Information). For each imaging channel, we first introduced a single kind of imager strands into the samples and performed imaging. Subsequently, the buffer was replaced with an imaging cocktail comprising equimolar of all three imager strands and conducted imaging in situ. As illustrated in Figure 1b, for all the channels, the localized spots obtained from single and mixed imagers were almost completely overlapped, indicating that the three imager strands that we chose did not interact with each other. Furthermore, to verify whether potential nonspecific binding events were overlooked due to spectral discrepancies, we performed cross‐validation experiments using three imaging strands conjugated with homologous fluorophores (P1‐AF647, P2‐AF647 and P3‐AF647). As shown in the Figure S4 (Supporting Information), only the complementary docking strand–imager strand pairs exhibited observable binding–dissociation events, whereas virtually no specific binding events were detected in mismatched combinations.

### 2.3 Binding Time Multiplexing

**Figure 2:**
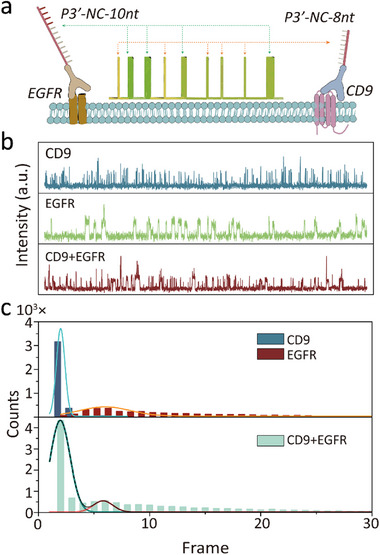
Multiplexed single‐molecule imaging of exosomal biomarkers using binding time as a kinetic parameter. a) Schematic illustration of the strategy utilizing 8 nt (CD9) and 10 nt (EGFR) hybridization lengths to distinguish between CD9 and EGFR based on distinct binding durations. b) Representative kinetic traces showing short‐duration binding events for CD9, long‐duration events for EGFR, and mixed events when both markers are co‐localized on A549 exosomes. c) Binding time distributions for CD9 and EGFR, demonstrating clear separation and minimal overlap, both when markers are present individually (top) and concurrently (bottom). The binding‐time distribution was derived from a gaussian fit.

Different binding times can also be used as a multiplexing parameter. We tested various DNA hybrization lengths, and found that 8 and 10 nt hybrization exhibited binding times with desirable durations and are distinct from each other. Then, we conjugated CD9 and EGFR antibodies to different docking strands to induce 8 (CD9) and 10 nt (EGFR) hybridization with a common imager strand (**Figure** **2**a), and used them to label A549 cell exosomes. As shown in Figure 2b, when there is only CD9 or EGFR on an exosome, the kinetic traces contain only short or long duration binding events, while when CD9 and EGFR occurs concurrently, short and long duration events are mixed. As illustrated in Figure 2c, the binding times of each marker formed two distinct distributions with minimal overlap, which is resolvable both when the markers occur separately (top) and concurrently (bottom) on an exosome. A threshold of 4 frames was chosen based on the binding time histogram to calculate the frequency of short and long duration events (Figure S5, Supporting Information). Then the frequencies were used to calculate the copy number (*N_s_ and N_l_*) and the expression percentage of the corresponding markers. The analysis results of CD9 and EGFR were consistent the result from nano‐flow cytometry, which further validated this method (Figure S6a,b, Supporting Information).

### 2.4 Photo‐Cleavage Response Multiplexing

**Figure 3:**
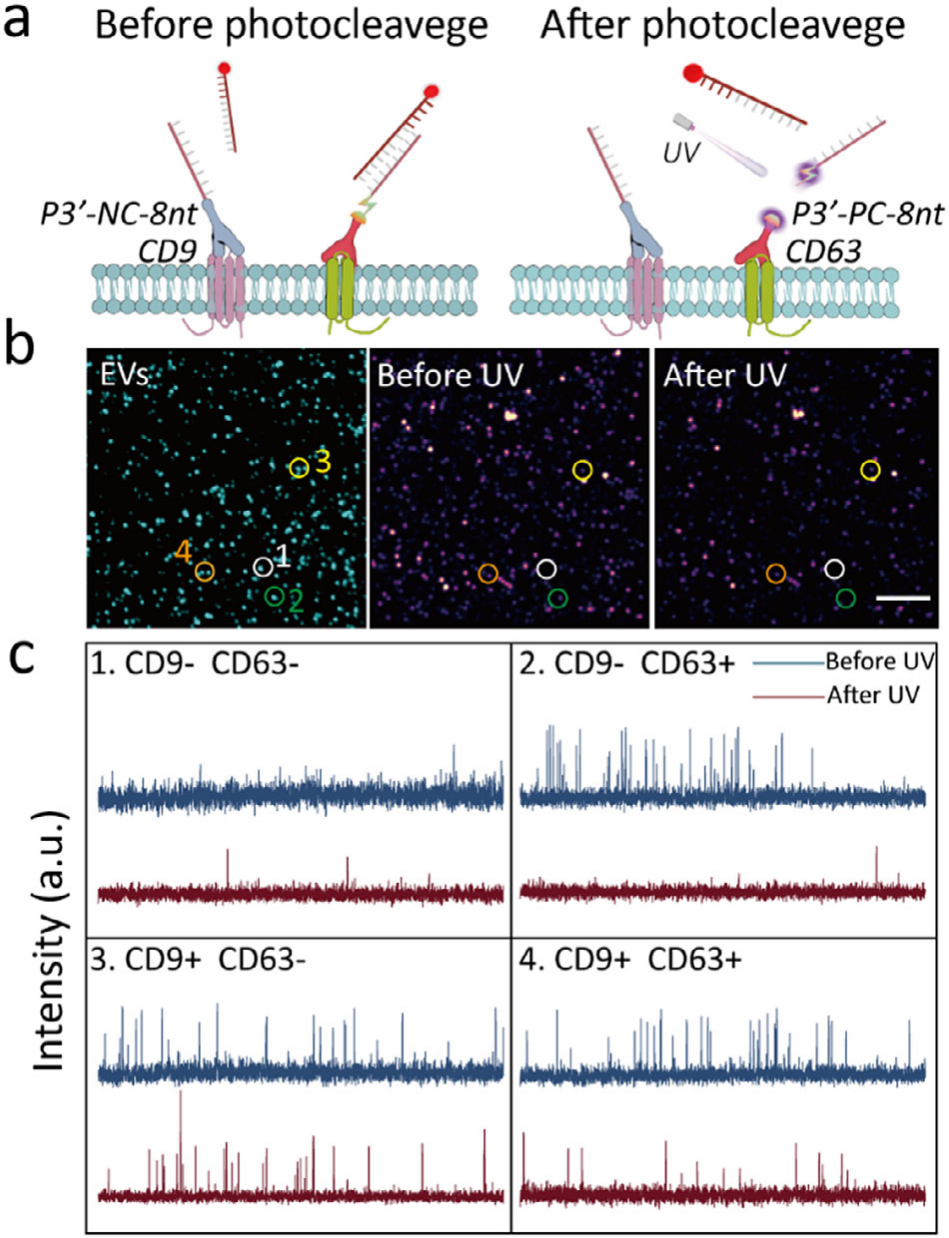
Multiplexed Single‐molecule imaging of exosomal biomarkers using UV‐induced frequency change. a) Schematic illustration of the principle for distinguishing CD9 and CD63 on A549 exosomes by UV‐induced cleavage of DNA docking strands b) Localization maps of exosomes (left), CD9 and CD63 before UV‐induced cleavage (middle), CD9 alone after UV exposure. c) Representative binding dynamics traces of exosomes with varying CD9 and CD63 surface marker expression, before and after UV‐induced cleavage. Scale bars, 5 µm.

The basic principle of this approach is that if an antibody‐DNA conjugate is responsive to external stimuli (UV light), then we can measure the binding frequency before and after applying the stimuli to resolve its information (**Figure** **3**a). To validate this method, we conjugated two antibodies (CD9, CD63) with the same DNA strand using non‐cleavable and photo‐cleavable linkers. According to our test, UV excitation can efficiently cleave 90% of the photo‐cleavable linkers, and at the same time does not affect the non‐cleavable linkers and the fluorophores (Figures S7–S9, Supporting Information). The CD9, CD63 antibody‐DNA conjugates were used to label A549 exosomes. As shown in Figure 3b, before UV exposure, CD9 and CD63 were undifferentiated and imaged simultaneously, after UV light, the DNA strands on the CD63 antibodies were cleaved and only CD9 were observed. At the single exosome level, we observed 4 different situations: when neither marker was presented, there was almost no binding event before or after cleavage; for exosomes with only CD63, no binding events were detected post‐cleavage; for exosomes with only CD9, the frequency of binding events did not change before and after cleavage; for exosomes expressing both CD9 and CD63, binding dynamics retained but was decreased in frequency after UV exposure (Figure 3c). Based on the binding frequency change, we can calculate two copy number of an exosome: one before (*N_b_*) and one after photo‐cleavage (*N_a_*), which correspond to the total copy number of CD9 and CD63, and the copy number of CD9, respectively. Then, it is straightforward to obtain CD63 copy number by subtraction. The results from this approach were also compared with the ones from nano‐flow cytometry for consistency (Figure S6a,c, Supporting Information).

### 2.5 Combination of the Multiplexing Modes

**Figure 4:**
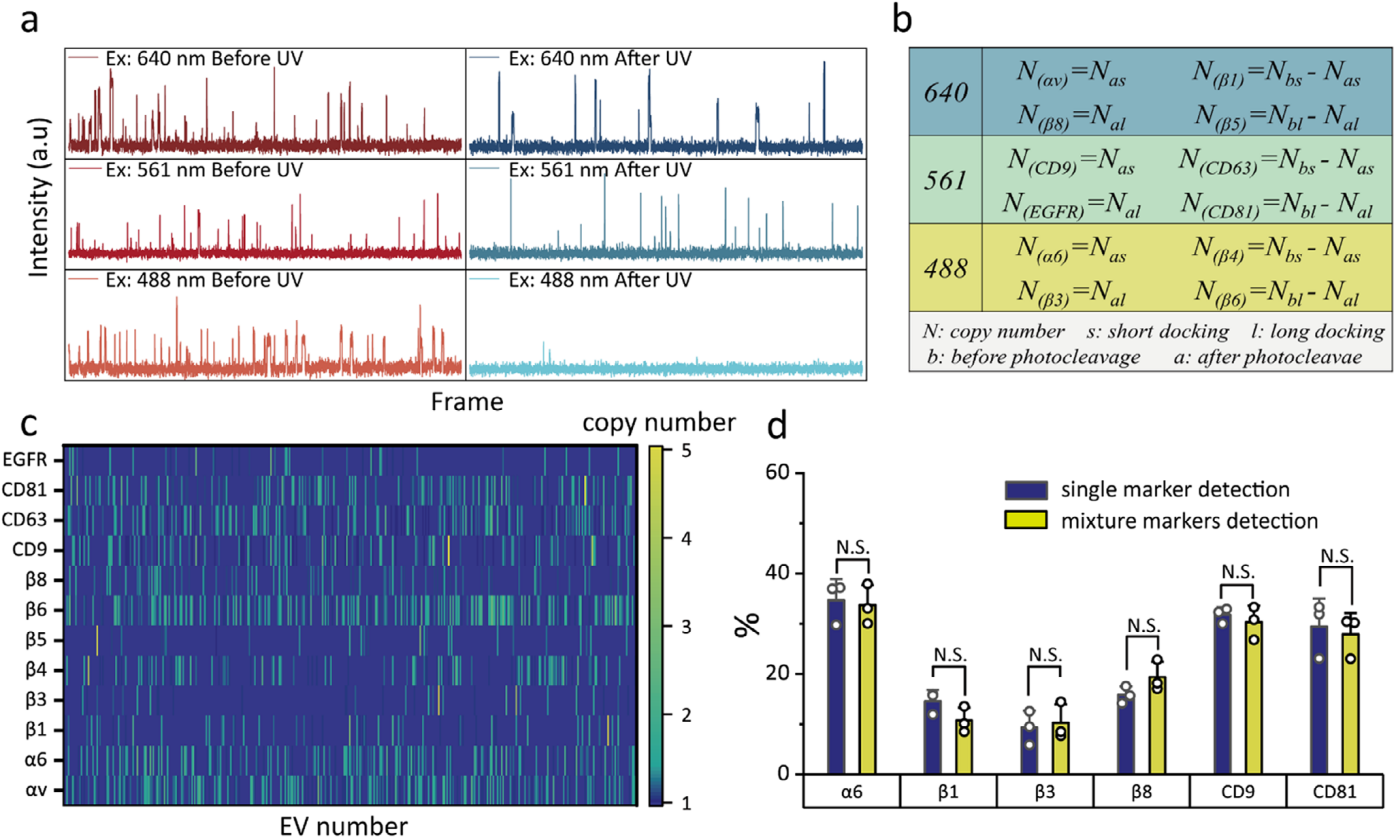
Multiplexed Single‐molecule Imaging of exosomal biomarkers using orthogonal combinations of imaging wavelength, binding time, and photo‐cleavable response. a) Representative binding dynamics of a single exosome across different imaging channels before and after photo‐cleavage. b) Mathematical formulas used to calculate the copy numbers of various markers detected on exosomes. c) A heatmap containing the digital protein expression profile of all the exosomes. d) Comparison of protein expression percentages obtained from 12‐plex DNA‐PAINT and single‐target DNA‐PAINT. Data are from three independent experiments and are presented as mean ± SD. Significance was determined by Tukey's test: ^*^ *p* <0.05, ^**^ *p* <0.01, ^***^ *p* <0.001, ^****^ *p* <0.0001; N.S., not significant.

After confirming the accuracy and detection consistency of each multiplexed assay, the three methods were orthogonally combined (Figure S10, Supporting Information). Since imaging wavelength (3×), binding time (2×) and photo‐cleavage response (2×) are orthogonal parameters, in principle, combining these multiplexing modes allows simultaneous imaging of up to 12 (3 × 2 × 2) surface markers on single exosomes. 4 markers can be resolved for each imaging channel. For each color, the kinetic traces can be divided into two parts, which correspond to before and after photo‐cleavage, respectively, and each part contains two types of binding events, i.e., short and long duration events (**Figure** **4**a). The short and long duration events before and after photo‐cleavage each can yield a copy number: *N_bs_*, *N_bl_*, *N_as_*, *N_al_*. As discussed above, the copy number determined before photo‐cleavage is the sum of two markers. Therefore, the actual copy numbers of the four markers are given by *N_as_*, *N_al_*, *N_bs_‐N_as_*, *N_bl_‐N_al_*, respectively. To demonstrate the concept, we selected eight integrin isoforms (αv, α6, β1, β3, β4, β5, β6, β8) and four common exosome markers (CD9, CD63, CD81, EGFR) as targets for imaging of HT29 cell exosomes (detailed workflow shown in Figure S11, Supporting Information; integrin antibody information shown in Methods and Figure S12, Supporting Information; DNA sequence information shown in Table S3, Supporting Information). Figure 4b shows assignment of each marker and provides formulas for their copy number calculation.

Using these formulas, we obtained a list containing the digital protein expression profile of all the exosomes in the imaging area (Figure 4c). To assess whether multiplex labeling influences analysis results, we compared the protein expression percentages obtained from twelve‐plex DNA‐PAINT with the results from single‐target DNA‐PAINT. As illustrated in Figure 4d and Figure S13 (Supporting Information), there was no significant difference in the marker expression rates and copy number, confirming the reliability of the method.

### 2.6 Profiling the Expression Landscape of Integrin Isoforms on Cellular Exosomes

We used this method to profile integrin isoforms on various types of exosomes with different lung‐targeting behavior. αv and α6 integrins are suspected to promote lung metastasis of various types of cancer, which are the focus of the study. Since integrin isoforms exhibit complex interactions that α and β isoforms can form heterodimers, it is also necessary to characterize the β isoforms that can bind with αv, α6. Therefore, a total of 8 types of integrins were characterized, namely, αv and its partner β3, β5, β6, β8, and α6 and its partner β1, β4.

**Figure 5:**
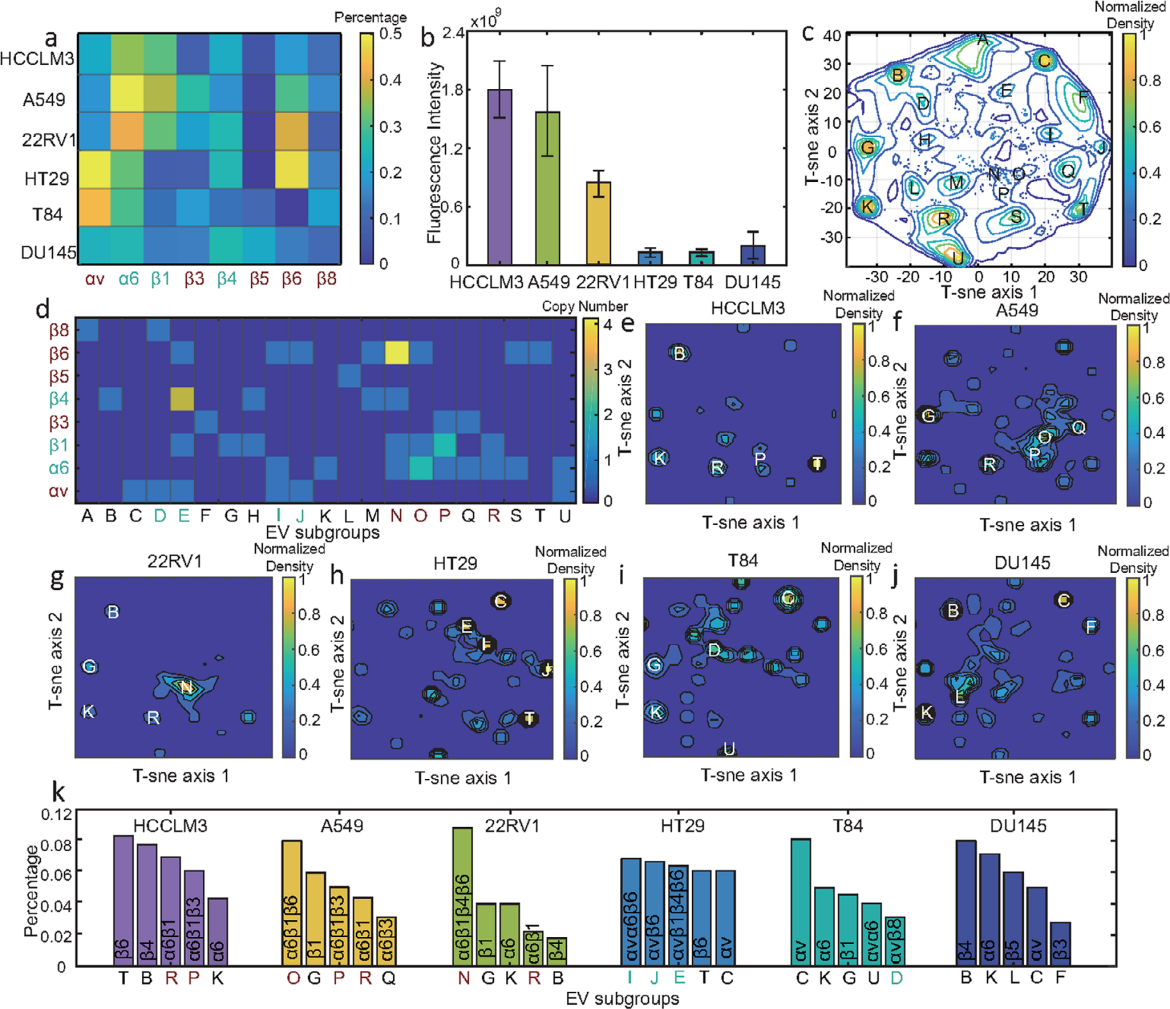
Integrin expression profiles of cellular exosomes with varying degree of lung tropism. a) A heatmap illustrating α and β integrins expression percentages across six types of exosomes. b) Mean fluorescence intensity of lung‐tropic exosomes in mouse lungs, *n* = 5 technical replicates. Data are presented as mean ± SD. c) t‐SNE scatter plot illustrating subgroups of all exosomes based on integrin expression profiles. d) The integrin expression profiles of different subpopulations in 5c. Subpopulations with paired αv, α6, and unpaired integrins are marked with cyan, red, and black letters, respectively. e–j) Normalized t‐SNE heatmaps of exosomes derived from different cell types, with the top five subpopulations highlighted with letters. k) Top five exosomal subpopulations of each cellular exosomes and their integrin expression profiles. Subpopulations with paired αv, α6, and unpaired integrins are marked with cyan, red, and black letters, respectively. Analysis of exosomal subpopulations was performed using a custom MATLAB script.

As shown in **Figure** **5**a, we selected six types of cellular exosomes (HCCLM3, A549, 22RV1, HT29, T84, DU145) based on their expression profile of αv, α6 integrins. Three types of exosomes primary express integrin α6 (HCCLM3, A549, 22RV1), two types of exosomes primary express integrin αv (HT29, T84), and one kind of exosome exhibits balanced expression of αv, α6 integrins (DU145). To compare the lung‐targeting effect of the exosomes, 100 mL of 10^10^ particle ˑ mL^−1^ of each type of exosomes stained with membrane dye were injected into mice from their tails. After 48 h, the mice were sacrificed to evaluate the fluorescence signals in the lungs. HCCLM3, A549, 22RV1 exosomes exhibited clear lung accumulation, while for HT29, T84, DU145 exosomes, no obvious fluorescence signal was observed (Figure S14, Supporting Information). Quantitative analysis showed that, among the lung‐tropic exosomes, HCCLM3 and A549 exosomes showed the stronger targeting effect with average fluorescence intensity almost twice as high as that of the 22RV1 exosomes (Figure 5b). The lung targeting performance of HCCLM3 exosomes perhaps is not surprising as it is a hepatocellular carcinoma line was established from a lung metastasis and possesses high lung‐metastatic potential. While the potent lung targeting of A549 exosomes is attributable to the “nest effect” expected of their lung cancer origin. However, we will show below that, at the molecular level, such effect is related to the distribution of integrins among individual exosomes.

To explore the molecular origin of the different lung‐targeting behaviors, we performed colocalization analysis of integrin isoforms and used t‐SNE algorithm to separate different subpopulations. For all the exosomes combined, ≈21 major clusters, which correspond to 21 key subgroups, can be obtained from the t‐SNE plot (Figure 5c). The integrin expression profiles of different subpopulations are shown in Figure 5d. The results showed that integrin isoforms are heterogeneously expressed with low copy numbers on single exosomes, and most of the exosomes contained mis‐paired and non‐paired integrins. The t‐SNE plot of different types of exosomes are shown in Figure 5e–j, with the top 5 largest subgroups highlighted with letters. The integrin compositions of the top 5 subgroups are shown in Figure 5k. Among the exosomes, HCCLM3, A549 and 22RV1 exosomes contain major subpopulation with paired α6 heterodimer, HT29 exosomes contain major subgroups with paired αv, and T84, DU145, contain mostly unpaired integrins.

By comparing these results with the lung targeting behavior, we concluded that integrin α6 likely plays an important role in exosomes lung tropism. This conclusion was further supported by in vivo integrin α6 blockade experiments in mice, in which functional inhibition of α6 led to a significant reduction in exosome lung tropism (Methods and Figure S15, Supporting Information). Moreover, control experiments in tumor‐bearing mice confirmed that the lung targeting mechanism mediated by integrin α6 is independent of the influence of the primary tumor (Methods and Figure S16, Supporting Information). At the single exosome level, since formation of hetero‐dimers are required for integrins to perform the adhesive function, colocalization of integrin α6 and the pairing β isoforms on individual exosomes can also affect the lung targeting behavior. This is consistent by our observation that HCCLM3 and A549 exosomes, which contained a notably larger subpopulation with paired α6 integrins than 22RV1 exosomes, showed stronger lung targeting effect. In some cellular exosomes, we observed a substantial fraction with non‐paired integrins. Since the α and the β isoforms are non‐covalently bonded, integrin subunits exhibit dynamic association, dissociation, and homo‐oligomerization during transmembrane sign transduction.^[^ ^37^, ^38^, ^39^, ^40^ ^]^ While previous researches have linked α6 integrin with lung‐tropism, little was known about how α_6_ integrin and its β partners are distributed among individual exosomes, in particular, whether α6 integrins mostly exist as monomers or paired dimers.^[^ ^5^ ^]^ Our study addressed this question and showed that exosomes with similar integrin α6 expression percentage can have vastly different pairing behavior at the single exosome level, and as a result showing different lung‐targeting effect.

## 3 Conclusion

Efficient profiling of large number of exosomal proteins at single‐molecule resolution is technically challenging. Current single‐molecule methods either suffer from limited multiplexing capability or require multi‐round staining and have low throughput. To overcome these limitations, we developed a single‐step, highly multiplexed single‐molecule imaging method based on DNA‐PAINT, which integrate spectral multiplexing, binding duration multiplexing, and photo‐cleavage response multiplexing. This innovative approach enables simultaneous quantification of up to 12 distinct exosomal protein markers at single‐copy resolution. Using this method, we characterized integrin expression profiles on various types of cell‐derived exosomes with different lung‐targeting behavior. We observed that exosomes with major subpopulations containing paired α6 dimer showed observable lung accumulation, while exosomes with primary αv dimer or unpaired integrins showed almost no lung targeting behavior, suggesting the important role of paired α6 dimer in lung tropism of exosomes. Overall, this work represents a systematical study of integrins on lung‐tropic exosomes at the single‐molecule level, which provides valuable implications to the development of exosome‐based biopsies and therapies.

## Conflict of Interest

The authors declare no conflict of interest.

## Supporting information



Supporting Information

## Data Availability

The data that support the findings of this study are available from the corresponding author upon reasonable request.

## References

[1] C. Théry , L. Zitvogel , S. Amigorena , Nat. Rev. Immunol. 2002, 2, 569. 12154376 10.1038/nri855

[2] G. Raposo , W. Stoorvogel , J. Cell. Biol. 2013, 200, 373. 23420871 10.1083/jcb.201211138 PMC3575529

[3] Y. Xiao , T. Driedonks , K. W. Witwer , Q. Wang , H. Yin , J. Extracell. Vesicles 2020, 9, 1793515. 32944182 10.1080/20013078.2020.1793515 PMC7480420

[4] Z. B. Li , K. Z. Guo , Z. T. Gao , J. Y. Chen , Z. Y. Ye , M. H. Cao , S. E. Wang , Y. D. Yin , W. W. Zhong , Sci. Adv. 2024, 10, adh8689. 10.1126/sciadv.adh8689 PMC10901469 38416840

[5] A. Hoshino , B. Costa‐Silva , T.‐L. Shen , G. Rodrigues , A. Hashimoto , M. Tesic Mark , H. Molina , S. Kohsaka , A. Di Giannatale , S. Ceder , S. Singh , C. Williams , N. Soplop , K. Uryu , L. Pharmer , T. King , L. Bojmar , A. E. Davies , Y. Ararso , T. Zhang , H. Zhang , J. Hernandez , J. M. Weiss , V. D. Dumont‐Cole , K. Kramer , L. H. Wexler , A. Narendran , G. K. Schwartz , J. H. Healey , P. Sandstrom , et al., Nature 2015, 527, 329. 26524530 10.1038/nature15756 PMC4788391

[6] Z. L. Li , C. Liu , Y. C. Cheng , Y. K. Li , J. Q. Deng , L. X. Bai , L. L. Qin , H. L. Mei , M. Zeng , F. Tian , S. H. Zhang , J. S. Sun , Sci. Adv. 2023, 9, ade2819. 10.1126/sciadv.ade2819 PMC10121168 37083528

[7] B. Q. Lin , T. Tian , Y. Z. Lu , D. Liu , M. J. Huang , L. Zhu , Z. Zhu , Y. L. Song , C. Y. Yang , Angew. Chem., Int. Ed. 2021, 60, 7582. 10.1002/anie.202015628 33382182

[8] W. S. Zheng , S. M. LaCourse , B. F. Song , D. K. Singh , M. Khanna , J. Olivo , J. Stern , J. N. Escudero , C. Vergara , F. F. Zhang , S. B. Li , S. Wang , L. M. Cranmer , Z. Huang , C. M. Bojanowski , D. R. Bao , I. Njuguna , Y. T. Xiao , D. C. Wamalwa , D. T. Nguyen , L. Yang , E. Maleche‐Obimbo , N. Nguyen , L. L. Zhang , H. Phan , J. Fan , B. Ning , C. Z. Li , C. J. Lyon , E. A. Graviss , et al., Nat. Biomed. Eng. 2022, 6, 979. 35986185 10.1038/s41551-022-00922-1 PMC9391224

[9] Q. Zhou , X. M. Niu , Z. Zhang , K. O'Byrne , A. Kulasinghe , D. Fielding , A. Möller , A. Wuethrich , R. J. Lobb , M. Trau , Adv. Sci. 2024, 11, 2041818. 10.1002/advs.202401818 PMC11434045 38885350

[10] S. Rana , S. Yue , D. Stadel , M. Zöller , Int. J. Biochem. Cell Biol. 2012, 44, 1574. 22728313 10.1016/j.biocel.2012.06.018

[11] R. J. Lobb , L. G. Lima , A. Möller , Cell Dev. Biol. 2017, 67, 3. 10.1016/j.semcdb.2017.01.004 28077297

[12] J. Kowal , G. Arras , M. Colombo , M. Jouve , J. P. Morath , B. Primdal‐Bengtson , F. Dingli , D. Loew , M. Tkach , C. Théry , Proc. Natl. Acad. Sci. USA 2016, 113, 968. 10.1073/pnas.1521230113 PMC4776515 26858453

[13] E. van der Pol , F. A. W. Coumans , A. E. Grootemaat , C. Gardiner , I. L. Sargent , P. Harrison , A. Sturk , T. G. van Leeuwen , R. Nieuwland , J. Thromb. Haemost. 2014, 12, 1182. 24818656 10.1111/jth.12602

[14] X. Liu , Z. Zong , M. Xing , X. Liu , J. Li , D. Liu , Nano. Lett. 2021, 21, 8817. 34609888 10.1021/acs.nanolett.1c03211

[15] J. Q. Deng , S. Zhao , K. Xie , C. Liu , C. G. Sheng , J. H. Li , B. Dai , S. Wan , L. L. Li , J. S. Sun , Angew. Chem., Int. Ed. 2025, 64, 202417165. 10.1002/anie.202417165 39513555

[16] P. Zhang , X. Zhou , M. He , Y. Shang , A. L. Tetlow , A. K. Godwin , Y. Zeng , Nat. Biomed. Eng. 2019, 3, 438. 31123323 10.1038/s41551-019-0356-9 PMC6556143

[17] W. Shen , K. Guo , G. B. Adkins , Q. Jiang , Y. Liu , S. Sedano , Y. Duan , W. Yan , S. E. Wang , K. Bergersen , D. Worth , E. H. Wilson , W. Zhong , Angew. Chem., Int. Ed. 2018, 57, 15675. 10.1002/anie.201806901 PMC6246790 30291794

[18] Y. Jiang , L. A. Andronico , S. R. Jung , H. Chen , B. Fujimoto , L. Vojtech , D. T. Chiu , Angew. Chem., Int. Ed. 2021, 60, 13470. 10.1002/anie.202103282 PMC8215978 33797851

[19] W. Zheng , S. M. LaCourse , B. Song , D. K. Singh , M. Khanna , J. Olivo , J. Stern , J. N. Escudero , C. Vergara , F. Zhang , S. Li , S. Wang , L. M. Cranmer , Z. Huang , C. M. Bojanowski , D. Bao , I. Njuguna , Y. Xiao , D. C. Wamalwa , D. T. Nguyen , L. Yang , E. Maleche‐Obimbo , N. Nguyen , L. Zhang , H. Phan , J. Fan , B. Ning , C. Li , C. J. Lyon , E. A. Graviss , et al., Nat. Biomed. Eng. 2022, 6, 979. 35986185 10.1038/s41551-022-00922-1 PMC9391224

[20] B. Ma , L. Li , Y. Bao , L. Yuan , S. Liu , L. Qi , S. Tong , Y. Xiao , L. Qi , X. Fang , Y. Jiang , Chem. Biomed. Imaging 2023, 2, 27. 39473463 10.1021/cbmi.3c00095 PMC11504620

[21] Y. Jiang , J. Zhang , S. R. Jung , H. Chen , S. Xu , D. T. Chiu , Angew. Chem. Int. Ed. 2023, 62, 202217889. 10.1002/anie.202217889 PMC9908834 36581589

[22] J. D. Spitzberg , S. Ferguson , K. S. Yang , H. M. Peterson , J. C. T. Carlson , R. Weissleder , Nat. Commun. 2023, 14, 1239. 36870999 10.1038/s41467-023-36932-z PMC9985597

[23] Y. Wang , J. B. Woehrstein , N. Donoghue , M. Dai , M. S. Avendaño , R. C. J. Schackmann , J. J. Zoeller , S. S. H. Wang , P. W. Tillberg , D. Park , S. W. Lapan , E. S. Boyden , J. S. Brugge , P. S. Kaeser , G. M. Church , S. S. Agasti , R. Jungmann , P. Yin , Nano. Lett. 2017, 17, 6131. 28933153 10.1021/acs.nanolett.7b02716 PMC5658129

[24] C. Chen , S. Zong , Y. Liu , Z. Wang , Y. Zhang , B. Chen , Y. Cui , Small 2019, 15, 1901014. 10.1002/smll.201901014 31478613

[25] Q. Zhou , J. Wang , Z. Zhang , A. Wuethrich , R. J. Lobb , M. Trau , Biosens. Bioelectron. 2024, 244, 115819. 37952322 10.1016/j.bios.2023.115819

[26] J. Schnitzbauer , M. T. Strauss , T. Schlichthaerle , F. Schueder , R. Jungmann , Nat. Protoc. 2017, 12, 1198. 28518172 10.1038/nprot.2017.024

[27] R. Yan , S. Moon , S. J. Kenny , K. Xu , Acc. Chem. Res. 2018, 51, 697. 29443498 10.1021/acs.accounts.7b00545

[28] Z. Zhang , S. J. Kenny , M. Hauser , W. Li , K. Xu , Nat. Methods 2015, 12, 935. 26280329 10.1038/nmeth.3528

[29] S. Moon , R. Yan , S. J. Kenny , Y. Shyu , L. Xiang , W. Li , K. Xu , J. Am. Chem. Soc. 2017, 139, 10944. 28774176 10.1021/jacs.7b03846

[30] R. Huang , E. K. Rofstad , J. Exp. Clin. Cancer Res. 2018, 37, 92. 29703238 10.1186/s13046-018-0763-x PMC5924434

[31] H. Liu , D. C. Radisky , D. Yang , R. Xu , E. S. Radisky , M. J. Bissell , J. M. Bishop , Cell Biol. 2012, 14, 567. 10.1038/ncb2491 PMC3366024 22581054

[32] Y. Jiang , J. McNeill , Chem. Rev. 2016, 117, 838. 28029769 10.1021/acs.chemrev.6b00419

[33] Y. Jiang , H. Chen , X. Men , Z. Sun , Z. Yuan , X. Zhang , D. T. Chiu , C. Wu , J. McNeill , Nano. Lett. 2021, 21, 4255. 33733782 10.1021/acs.nanolett.1c00405 PMC10279485

[34] R. Jungmann , M. S. Avendano , M. Dai , J. B. Woehrstein , S. S. Agasti , Z. Feiger , A. Rodal , P. Yin , Nat. Methods 2016, 13, 439. 27018580 10.1038/nmeth.3804 PMC4941813

[35] R. Jungmann , M. S. Avendano , J. B. Woehrstein , M. Dai , W. M. Shih , P. Yin , Nat. Methods 2014, 11, 313. 24487583 10.1038/nmeth.2835 PMC4153392

[36] O. K. Wade , J. B. Woehrstein , P. C. Nickels , S. Strauss , F. Stehr , J. Stein , F. Schueder , M. T. Strauss , M. Ganji , J. Schnitzbauer , H. Grabmayr , P. Yin , P. Schwille , R. Jungmann , Nano. Lett. 2019, 19, 2641. 30864449 10.1021/acs.nanolett.9b00508 PMC6463241

[37] C. Mas‐Moruno , R. Fraioli , F. Rechenmacher , S. Neubauer , T. G. Kapp , H. Kessler , Angew. Chem., Int. Ed. 2016, 55, 7048. 10.1002/anie.201509782 27258759

[38] K. R. L. Markus Moser , R. Zent , R. Fässler , Science 2009, 324, 895. 19443776 10.1126/science.1163865

[39] T.‐L. Lau , C. Kim , M. H. Ginsberg , T. S. Ulmer , EMBO J. 2009, 28, 1351. 19279667 10.1038/emboj.2009.63 PMC2683045

[40] R. H. Li , C. R. Babu , J. D. Lear , A. J. Wand , J. S. Bennett , W. F. DeGrado , Proc. Natl. Acad. Sci. USA 2001, 98, 12462. 11606749 10.1073/pnas.221463098 PMC60076

