# Supplementary material for: Profiling of Integrin Isoforms on Lung‐Tropic Exosomes by Spectrally and Kinetically Multiplexed Single‐Molecule Imaging
Source: Adv Sci (Weinh). 2025 Dec 31;13(18):e15129. doi: 10.1002/advs.202515129 (PMC13042962; doi:10.1002/advs.202515129)
Supplement: Supplementary file 1 — Supporting Information [file ADVS-13-e15129-s001.docx]

# Supporting Information

**Profiling of Integrin Isoforms on Lung-tropic Exosomes by Spectrally and Kinetically Multiplexed Single-molecule Imaging**

Songlin Liu, ^#[a,b]^ Haixin Wang, ^#[b]^ Qin Shentu, ^[b]^ Liang Yuan, ^[b]^ Jiangshan Tie, ^[b]^ Li Li, ^[b]^ Rui Ai, ^[b]^ Bochen Ma, ^[b]^ Lubin Qi, *^[b]^ Yifei Jiang, *^[b]^ and Xiaohong Fang *^[a,b,c,d]^

[a] S. Liu, Prof. Dr. X. Fang

School of Chemistry and Materials,

University of Science and Technology of China,

Hefei, Anhui, 230026, P. R. China

Email address: xfang@iccas.ac.cn

[b] S. Liu, H. Wang, Q. Shentu, L. Yuan, J. Tie, L. Li, R. Ai, B. Ma, Prof. Dr. R. Qi, Prof. Dr.

Y. Jiang, Prof. Dr. X. Fang

Hangzhou Institute of Medicine (HIM), Chinese Academy of Sciences,

Hangzhou, Zhejiang, 310022, P. R. China

Email address: [jiangyf@ibmc.ac.cn](mailto:jiangyf@ibmc.ac.cn) qilubin@mail.sdu.edu.cn

[c] Prof. Dr. X. Fang

School of Molecular Medicine,

Hangzhou Institute for Advanced Study, UCAS

Hangzhou, 310024, P. R.China

[d] Prof. Dr. X. Fang

Beijing National Research Center for Molecular Sciences, Institute of Chemistry, Key Laboratory of Molecular Nanostructure and Nanotechnology, Chinese Academy of Science

Beijing, 100190, P. R. China

TABLE OF CONTENTS

[METHODS S2](#_Toc214054155)

[Functionalized modification of glass S2](#_Toc214054156)

[DNA labeling of antibodies S2](#_Toc214054157)

[Cell culture and exosome isolation S2](#_Toc214054158)

[PFO polymer dots preparation and bioconjugation S3](#_Toc214054159)

[DNA Tetrahedra Preparation S3](#_Toc214054160)

[Sample preparation of DNA nanostructure and exosomes S3](#_Toc214054161)

[TIRF microscope setups and imaging condition S4](#_Toc214054162)

[Data analysis S4](#_Toc214054163)

[Exosome labeling and in vivo tracking S5](#_Toc214054164)

[Integrin α6 blockade mouse model in vivo exosome tracking S5](#_Toc214054166)

[Tumor-bearing mouse model and in vivo exosome tracking S6](#_Toc214054165)

[Statistical analysis S6](#_Toc214054165)

[Antibody information S6](#_Toc214054166)

[SUPPORTING RESULTS AND DISCUSSION S7](#_Toc214054167)

Agarose gel electrophoresis of DNA tetrahedrons[. S7](#_Toc214054168)

[Determination of influx Rates Using DNA Nanostructures S7](#_Toc214054169)

[Laser channel crosstalk validation across different imager strands S8](#_Toc214054170)

[Assessment of nonspecific binding among different imager and docking strands pairs S9](#_Toc214054171)

[Binding-time distribution of different imager-Docking strand pairs S10](#_Toc214054172)

[Validation of binding time and PC-induce multiplexing via nanoflow cytometry S10](#_Toc214054173)

[Validation of photo-cleavable efficiency at the bulk level. S11](#_Toc214054174)

[Validation of photo-cleavable efficiency at the single-molecule level S11](#_Toc214054174)

[Validation of Control Group for Photo-Cleavage Response Multiplexing S12](#_Toc214054175)

[Cross-validation of consistency among different multiplexing modes S13](#_Toc214054177)

[Experimental workflow for Multiplexed Single-molecule Imaging S14](#_Toc214054178)

[Antibody Specificity Validation S14](#_Toc214054179)

[Comparison of copy number obtained from 12-plex DNA-PAINT and single-target DNA-PAINT. S18](#_Toc214054180)

[Representative fluorescence images of mouse lungs. S18](#_Toc214054181)

[Assessment of exosome enrichment in the lungs of integrin α6 blockade mice. S18](#_Toc214054181)

[Assessment of exosome enrichment in the lungs of tumor-bearing mice. S18](#_Toc214054181)

[Comparison of performances between this work and other single-exosome analysis methods. S18](#_Toc214054181)

[Influx rates of different imager strands and docking strands. S18](#_Toc214054181)

[DNA sequences for Imager strands and docking strands. S18](#_Toc214054181)

[DNA sequences for the synthesis of DNA tetrahedra S20](#_Toc214054183)

[REFERENCE S20](#_Toc214054184)

# METHODS

## Functionalized modification of glass

The PEGylated-biotin slides were prepared following the protocol outlined by Chandradoss et al.^[1]^ Glass slides were subjected to a sequential cleaning process, which included sonication in MilliQ water for 5 min (repeated three times), sonication in a 2% Alconox solution (242985, Sigma) for 15 min, and sonication in 100% acetone for 5 min (repeated three times). The slides were then rinsed three times with MilliQ water. To ensure thorough cleaning and hydroxylation, the slides were immersed in a piranha solution (7:3 H₂SO₄:H₂O₂) for 60 min. Following this, the slides were rinsed three times with HPLC ethanol and MilliQ water. Subsequently, the slides were incubated in an HPLC ethanol mixture containing 3% 3-aminopropyl triethoxysilane (706493, Sigma) and 5% acetic acid (45754, Sigma) for 30 min. After repeating the cleaning steps, the slide surfaces were passivated and biotinylated using polyethylene glycol (PEG) derivatives in a 0.1 M sodium bicarbonate solution, with a Biotin-PEG-SVA-5000 to PEG-SVA-5000 (Laysan Bio) ratio of 1:40. A 20 μL aliquot of the PEG solution was applied to the slide surface and incubated overnight under dark, humid conditions. Finally, the slides were washed three times with MilliQ water and dried using nitrogen gas.

## DNA labeling of antibodies

DNA labeling of antibodies was performed according to the protocol described by Ralf Jungmann et al.^[2]^ First, the 5′-thiolated DNA strand (Table S3) was reduced with dithiothreitol (646563, Sigma) and purified using NAP-5 columns. Next, the antibodies were crosslinked with either a maleimide-PEG-succinimidyl ester crosslinker (NC linker) (223221, Thermo) or a PC maleimide-NHS carbonate ester (PC linker) (HY-140140, MCE) in a 50× molar excess. Unreacted linker was removed using Amicon spin filters (50 kDa). The antibody-linker complex was then reacted with the reduced 5′-thiolated DNA strand at 4°C overnight. Finally, the DNA-labeled antibodies were purified using a fresh 50 kDa Amicon Ultra filter to remove unreacted DNA. Each antibody was typically coupled with two docking strands.

## Cell culture and exosome isolation

In this study, we employed six cell lines: HCCLM3 (RRID: CVCL_6832), A549 (RRID: CVCL_0023), HT29 (RRID: CVCL_0320), 22RV1 (RRID: CVCL_1045), DU145 (RRID: CVCL_0105), and T84 (RRID: CVCL_0555), All cell lines were obtained from Procell Life Science & Technology Co., Ltd (Wuhan, China) and authenticated by STR profiling to confirm identity. The cell lines were maintained under standard culture conditions to ensure absence of contamination. Cells were cultured in their respective growth media, supplemented with 10% fetal bovine serum (FBS) and 1% penicillin-streptomycin, with media replacement every 72 hours. The selection of cell lines was based on the integrin expression profiles of the derived exosomes, which was double checked by single-molecule imaging and western blot before the animal experiments. Once sufficient cell density was achieved after multiple passages, the cells were transferred to a serum-free, specialized exosome production medium for 36 h. The conditioned medium was then collected for exosome isolation.

The collected conditioned medium underwent a series of gradient centrifugation steps. First, it was centrifuged at 300 ×g for 10 min to remove dead cells, followed by a second centrifugation at 3000 ×g for 10 min to eliminate cellular debris. The resulting supernatant was filtered through a 0.22 μm pore-size filter and concentrated using an Amicon Ultra-15 100 kDa filter. The concentrated supernatant was loaded onto an agarose size-exclusion column equilibrated with DPBS. Fractions containing exosome were collected and further concentrated using an Amicon Ultra-4 100 kDa filter. The concentrated exosome solution was aliquoted and stored at -80 °C for subsequent experiments.

## PFO polymer dots preparation and bioconjugation

PFO polymer dots were used to label exosome membranes, and their preparation followed a method similar to that previously reported.^[3]^ Specifically, poly(9,9-dioctylfluorene) (PFO) and poly(styrene-co-maleic anhydride) (PSMA) were dissolved in tetrahydrofuran (THF) to achieve a concentration of 20 ppm. The PFO and PSMA solutions were mixed in a 5:1 volume ratio to prepare the precursor solution. Next, 1 mL of the precursor solution was rapidly mixed with 9 mL of water under sonication. The mixture was heated under a nitrogen atmosphere to evaporate the THF. The resulting solution was filtered through a 220 nm pore-size filter to obtain hydrophilic PFO polymer dots. The nanoparticles were characterized using dynamic light scattering (DLS), which revealed a particle size distribution centered around 28 nm. The excitation and emission spectra of PFO indicate that it can be efficiently excited by a 405 nm laser. This excitation wavelength minimizes spectral overlap with other imaging agents, preventing crosstalk and ensuring accurate signal detection.

PFO ploymer dots were conjugated to streptavidin via an EDC-catalyzed reaction. For the conjugation, 1 mL of PFO suspension was mixed with 20 μL of PEG (5% w/v), 20 μL of 1 M HEPES buffer, 20 μL of EDC (5 mg/mL) and 10μL streptavidin (1 mg/mL). After stirring the reaction for 4 h, the solution was centrifuged. using Amicon Ultra-0.5 100 kDa filter and diluted three times in HEPES buffer containing 0.2% BSA (w/v). The final solution was concentrated to 0.5 mL and stored at 4 °C。

## DNA Tetrahedra Preparation

DNA tetrahedra with 1, 2, 3, and 4 binding sites were synthesized using a self-assembly method.^[4]^ Each tetrahedron is formed by the hybridization of four single-stranded DNA molecules, and the corresponding sequences are provided in Table S4. The 5′ end of the A strand is modified with biotin for attachment to a streptavidin-coated substrate. Depending on the number of required binding sites, the other three strands may include one or more strands with binding sites. The four ssDNA strands were dissolved and mixed in equal molar amounts in a Tris-Mg buffer (10 mM Tris, 5 mM MgCl₂, pH 8.0). The mixture was heated to 95°C for 5 mins, immediately cooled to 4°C for 10 mins, and then allowed to hybridize at 4°C for at least 4 hours. Following the reaction, the nanostructures were visualized using agarose gel electrophoresis (w/v, 1.5%). The gel band containing the target DNA tetrahedra was excised, and a gel extraction kit (Yeasen, 19101ES50) was employed to purify the excised portion, thereby obtaining the pure DNA tetrahedral structures. Agarose gel electrophoresis confirmed that the purified DNA tetrahedra, engineered with varying numbers of binding sites, exhibited high purity (Figure S1).

## Sample preparation of DNA nanostructure and exosomes

In the sample preparation section, a circular incubation chamber synthesized from PDMS was securely fitted to the biotinylated glass substrate. 40 μL of 2.5 mg/mL streptavidin was added and incubated for 15 min, followed by three washes with DPBS.

*For DNA structure sample preparation:* 40 μL of biotinylated DNA nanostructures (10 pM) were added to the reaction chamber and incubated for 15 min. The chamber was then washed three times with DPBS.

*For exosome sample preparation:* First, the exosomes need to be functionalized with biotin. the concentrated exosomes (20 μL) were mixed with DSPE-PEG-Biotin (10 mg/mL, 4 μL) and DPBS (476 μL), followed by thorough mixing and reacting for 20 min to achieve biotin assembly on the surface of the exosomes. Subsequently, the biotinylated exosomes were purified to remove excess DSPE-PEG-Biotin using agarose size-exclusion chromatography. 40 μL of biotinylated exosomes were introduced into the reaction chamber and incubated for 15 min. The chamber was then rinsed three times with DPBS to remove exosomes that were not anchored to the substrate. The antibody-DNA conjugates were dissolved in DPBS to prepare an antibody diluent with a concentration of approximately 5 μg/mL. 40 μl antibody diluent were then introduced into the reaction chamber and incubated for 60 min, followed by washing with washing buffer 3 times. Finally, 40 μL of a 200× solution of streptavidin-PFO was added to the reaction chamber for 15 min to bind the biotin on the exosome surface. Subsequently, any excess streptavidin-PFO was removed by washing the chamber three times with DPBS.

## TIRF microscope setups and imaging condition

The imaging experiment was performed using a Total Internal reflection fluorescence microscope (TIRFM) based on an Olympus IX83 inverted microscope, applying an oil-immersion objective (100×, NA 1.49). The laser excitation wavelengths employed in the experiment were 405 nm (100 mW, Olympus), 488 nm (100 mW, Olympus), 561 nm (100 mW, Olympus), and 640nm (100 mW, Olympus) respectively. The laser beam was passed sequentially through multiband bandpass filters (89402x and ZET405/488/561/647m, Chroma Technology) and single-band bandpass filter (FF01-445/40-25, Semrock and ET520/20M, ET609/34M, ET690/50M, Chroma Technology). Imaging was performed using an sCMOS camera (primeBSI, Teledyne Photometrics) without additional magnification. This resulted in an effective pixel size of 130 nm after 2×2 binning.

The imager strands were dissolved in PBS supplemented with 100 mM NaCl to reach a final concentration of 2 nM. The resulting imaging solution was subsequently applied to the sample. A movie of successive frames was obtained for each sample with an exposure time of 100 ms and a lapse interval of 10 ms.

In experiments involving photo cleavage, a UV light source (365 nm) was positioned directly above the sample substrate. The sample was irradiated for 15 mins, after which image acquisition was continued.

In the experiments analyzing multiple exosomal markers, the excitation light sequence for movie acquisition was as follows: 640 nm, 561 nm, 488 nm, followed by UV light for photo-cleavage, and then again 640 nm, 561 nm, and 488 nm. Prior to each movie acquisition, a 405 nm laser was used to excite PFO to determine the positions of the exosomes. This initial step was crucial for subsequent manual correction of drift during the data analysis process. (Figure S11)

## Data analysis

**Determination of influx rates (**ξ**) using single-strand DNA**

The following steps were performed：

*Localization and Reconstruction*: Movies were analyzed using the localize and render module in Picasso software to reconstruct the positions of DNA nanostructures within the sample. ^[2]^

*Single-molecule Center Determination*: A custom MATLAB script was used to determine the center of each point in the reconstructed images. A 7×7 pixels region around each point center was defined as the region of interest (ROI).

*Extraction of DNA Binding Kinetics Traces:* We employed a custom-developed MATLAB program to extract binding kinetics trajectories for each ROI from videos. Specifically, our analysis encompassed the quantification of binding event numbers and the determination of associated binding durations for each binding event.

*Threshold Determination*: The binding-time distribution was used to establish a threshold for distinguishing between short and long DNA binding events.

*Influx Rate(ξ) Calculation*: The mean dark-state time (τ_d*_) were used to calculate *ξ* for different combinations of docking strands and imager strands under the same imaging conditions.

**Analysis of exosomal marker expression profiles**

Consists of the following steps:

*Drift Correction:* Each exosome detection sample comprises six video files, including three fluorescence channels with two video files each (before- and after-photocleavage). Prior to analysis, manual drift correction was performed using exosome localization images captured before each video sequence to mitigate positioning errors induced by sample drift during acquisition.

*Exosome Localization:* Exosome positions were identified based on fluorescence signals emitted by PFO. A 7×7 pixels ROI centered on single-molecule spots was defined to precisely determine exosome locations.

*Extraction of DNA Binding Kinetic Traces:* DNA binding kinetic traces within each ROI were extracted from each videos using a custom Matlab script for subsequent analysis.

*Analysis of DNA Binding Kinetic Traces:* For a given laser channel, DNA binding kinetic traces were analyzed before and after UV cleavage. The number of binding events and their corresponding durations were quantified. Binding events were classified as short or long using a predefined threshold, enabling the calculation of short DNA average dark-state times (*τ_d*(bs)_* and *τ_d*(as)_*) and long DNA average dark-state times (*τ_d*(bl)_* and *τ_d*(al)_*) before and after photo-cleavage.

*Quantification of DNA Binding Sites and Marker Copy Numbers:* The average dark-state times (*τ_d*(bs)_, τ_d*(bl)_, τ_d*(as)_*, and *τ_d*(al)_*) and the parameter *ξ* derived from ssDNA standards were used in the formula N = (ξ × τ_d*_)^−1^ to determine the number of DNA binding sites. Since each antibody contains two binding sites, the total number of binding sites was divided by 2 to obtain *N_as_*, *N_al_*, *N_bs_*, and *N_bl_*. The corresponding marker copy numbers for a single channel were calculated as *N_as_*, *N_al_*, *N_bs_-N_as_*, and *N_bl_-N_al_*. A marker is considered positive if *N* >1, indicating its copy number as N; conversely, if *N* <1, the marker is deemed non-expressed. This methodology was uniformly applied to all other channels.

## Exosome labeling and in vivo tracking

Exosomes isolated from HCCLM3, 22RV1, DU145, HT29, A549 and T84 cell lines were quantified by nanoparticle tracking analysis (NTA) and adjusted to 1×10¹⁰ particles/100 μL in DPBS. For membrane labeling, 500 μL aliquots of each exosome preparation were incubated with 1 μL DiR fluorescent dye (DiRBB-441932) at 37°C for 1 h with gentle agitation, followed by three purification cycles using 100 kDa molecular weight cutoff filters (3,500 × g, 5 min per cycle) with DPBS washes to remove unincorporated dye. The labeled exosomes were reconstituted to the original concentration (1×10¹⁰ particles/100 μL) in DPBS. Four-to-six-week-old female nude mice (n=5 per group) received 100 μL of labeled exosome suspension via tail vein injection. Longitudinal in vivo imaging was performed using an IVIS Lumina III system at 0, 6, 12, 24, 36, and 48 h post-injection under isoflurane anesthesia (2-3% in oxygen). At the 48 h endpoint, mice were euthanized by CO₂ asphyxiation, followed by ex vivo imaging of lung organs. (All animal experiments were approved by the Hangzhou Medical Research Institute, Chinese Academy of Sciences, AP2024-08-0137)

## Integrin α6 blockade mouse model and in vivo exosome tracking

Exosomes from A549 and 22RV1 cells were quantified by nanoparticle tracking analysis, adjusted to 1×10¹⁰ particles/100 µL, and 500 µL aliquots of each were labeled with 1 µL DiR dye during 1 h of incubation at 37 °C in the dark. Unbound dye was subsequently removed using a 100 kDa ultrafiltration device by centrifugation at 3500 ×g for 5 min, followed by three DPBS washes. The labeled exosomes were finally resuspended in DPBS at the original concentration of 1×10¹⁰ particles/100 µL. Female nude mice aged 4–6 weeks were randomly divided into two groups (n = 5 per group) and administered 100 µL of the labeled exosome suspension derived from either A549 or 22RV1 cells via tail vein injection. Concurrently, an integrin alpha 6 antibody (5 µg/mL, Bio-Techne, MAB13501) was administered via intraperitoneal injection. Finally, at 48 hours post-injection, all mice were euthanized by CO₂ asphyxiation, and lung tissues were collected for ex vivo fluorescence imaging.

## Tumor-bearing mouse model and in vivo exosome tracking

A subcutaneous xenograft model was established by inoculating A549 cells (5×10⁵ per mouse) into the right flank of mice, followed by a 7-day tumor development period. Exosomes were quantified via nanoparticle tracking analysis to a concentration of 1×10¹⁰ particles/100 µL, and 500 µL aliquots of exosomes derived from A549 and HT29 cells were prepared. The exosomes were then labeled by incubation with 1 µL of DiR fluorescent membrane dye at 37 °C for 1 hour in the dark. Following incubation, unincorporated dye was removed by centrifugation at 3500 ×g for 5 minutes using a 100 kDa ultrafiltration device; The labeled exosomes were resuspended in DPBS and adjusted to a final concentration of 1×10¹⁰ particles/100 µL. This preparation was administered intravenously via the tail vein to the A549 tumor-bearing mice. Finally, at 48 hours post-injection, the mice were euthanized by CO₂ asphyxiation, and lung tissues were harvested for ex vivo imaging.

## Statistical analysis

Statistical analyses were performed using Origin software (version 2025 & 2026), while image analyses were conducted with ImageJ. Single-vesicle fluorescence kinetics and exosome subpopulations were analyzed using custom MATLAB scripts which employ single-molecule localization and t-SNE clustering algorithms reported previously.^[5-6]^ Data are presented as mean ± standard deviation (SD). For comparisons among multiple groups, one-way ANOVA followed by Tukey's post-hoc test was applied. A p-value of less than 0.05 was considered statistically significant.

## Antibody Information

| Antibody Name | manufacturer | Cat. No. |
| --- | --- | --- |
| CD9 Recombinant monoclonal antibody | Proteintech | 84142-1-RR |
| CD63 Polyclonal antibody | Proteintech | 25682-1-AP |
| CD81 Monoclonal Antibody | ThermoFisher | MA5-32333 |
| EGFR Monoclonal antibody | Proteintech | 66455-1-Ig |
| Integrin αv antibody | Proteintech | 84883-5-RR |
| Integrin α6 antibody | Proteintech | 84463-3-RR |
| Integrin β1 antibody | Proteintech | 12594-1-AP |
| Integrin β3 antibody | Proteintech | 83053-4-RR |
| Integrin β4 antibody | Proteintech | 21738-1-AP |
| Integrin β5 antibody | Proteintech | 28543-1-AP |
| Integrin β6 antibody | Cell Signaling | E4M9P |
| Integrin β8 antibody | Abcam | EPR27246-85 |
| Goat anti-rabbit IgG Secondary Antibody | MiaoBo Bio | MBT214513 |

# **SUPPORTING RESULTS AND DISCUSSION**


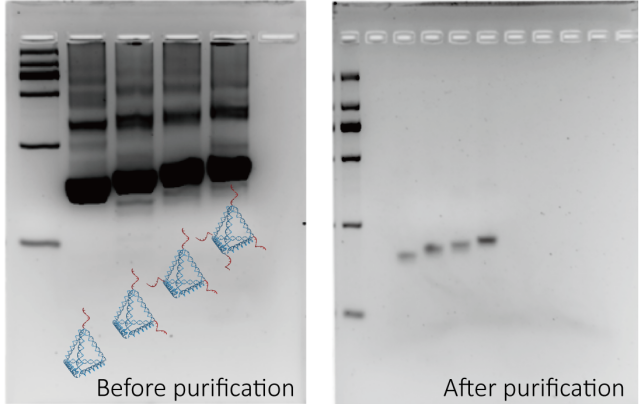


## Figure S1. Agarose gel electrophoresis of DNA tetrahedrons with varying numbers of binding sites before (left) and after (right) purification.

## Determination of influx Rates Using DNA Nanostructures

To validate the feasibility of quantifying binding sites through DNA binding-dissociation kinetics and to ensure consistent influx rates across different samples under identical imaging conditions, we synthesized DNA tetrahedral structures with 1, 2, 3, and 4 binding sites. These structures were immobilized on streptavidin-coated substrate via biotinylated DNA strands which are equipped with an additional poly-T tails to reduce steric hindrance. Therefore, each streptavidin can bind to three DNA tetrahedrals. We then added 2 nM of imager strands in 100 mM NaCl solution and collected intensity-time trajectories.

As shown in Figure S2a, no significant localization points were observed after reconstruction analysis in the control group without DNA tetrahedra. In contrast, when the substrate was assembled with DNA tetrahedra, frequent binding-dissociation events between the imager and docking strands on the tetrahedra resulted in a high density of localization points in the reconstructed images, confirming effective recognition and binding-dissociation events (Figure S2b).

We subsequently collected trajectories for tetrahedra with varying numbers of binding sites and analyzed the binding-dissociation events. The distribution of these event frequencies is presented in Figure S2c. Given the tetrahedral geometry of SA and three unoccupied biotin-binding sites per molecule in the presence of excess DNA nanostructures, each SA presents extended binding site clusters numbering 3, 6, 9, or 12 sites depending on spatial arrangement. As the number of binding sites increased, so did the frequency of binding-dissociation events. A linear relationship was observed between the number of binding sites and the frequency of binding-dissociation events (Figure S2d), demonstrating that DNA binding-dissociation kinetics can be used to quantify binding sites under consistent imaging conditions.


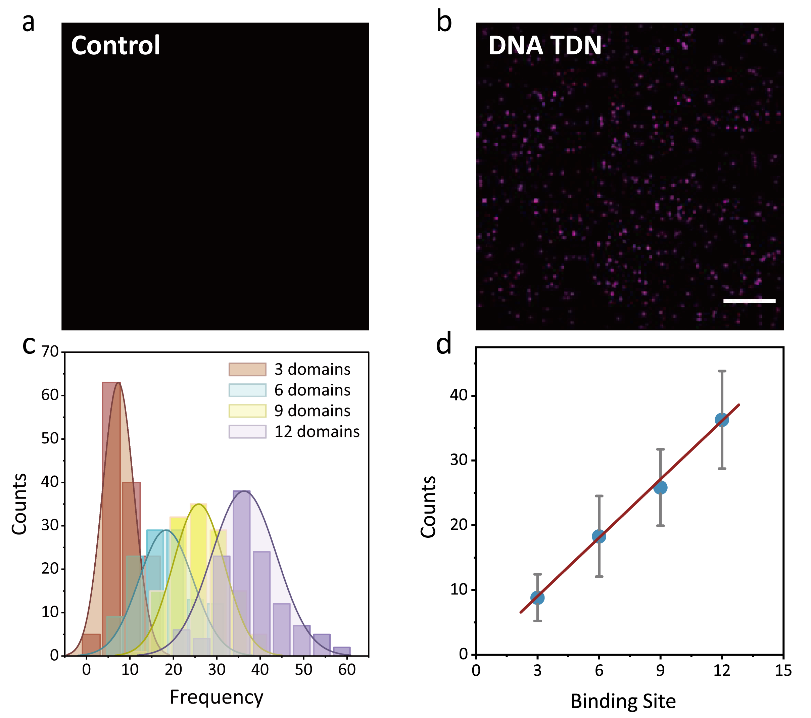


**Figure S2.** The localization sites of the control group without DNA tetrahedra (a) and the experimental group with DNA tetrahedra (b). (c) Frequency distribution of DNA binding and dissociation events for tetrahedra with different numbers of binding sites. (d) Linear correlation between tetrahedron binding sites and binding event frequencies. Each DNA tetrahedron contained over 300 distinct localization sites, with data presented as mean ± SD. Scale bars, 10 µm


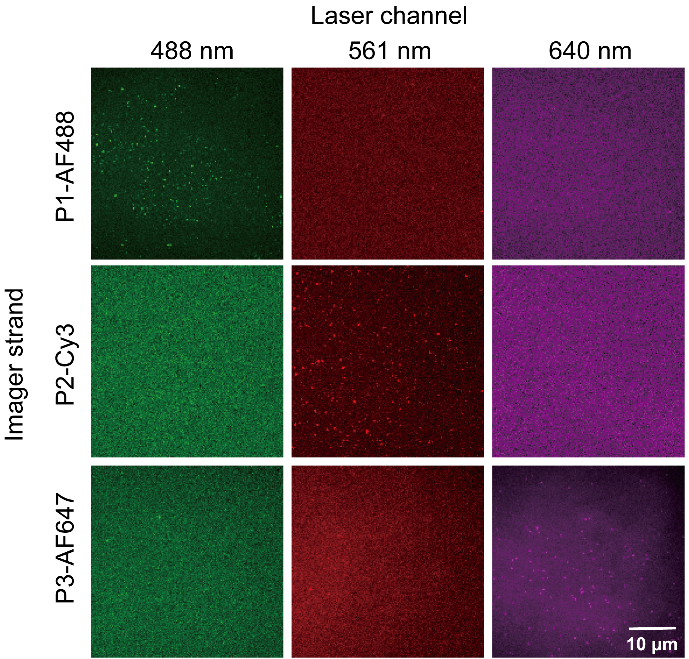


## Figure S3. Laser channel crosstalk validation across different imager strands. Single-frame fluorescence spot images of imager strands labeled with distinct fluorophores under different excitation wavelengths. (Experimental conditions: Three A549 exosome samples were prepared. To each sample, 40 μL of 5 μg/mL CD9 antibody-DNA conjugates (with docking strands P1’-NC-8NT, P2’-NC-8NT, and P3’-NC-8NT, respectively) were added to capture CD9 on the exosome surface. Subsequently, 2 nM of P1-AF488, P2-Cy3, and P3-AF647 were added to the three sample pools, respectively. Each sample was imaged under three different excitation wavelength conditions.) Scale bars, 10 µm

## Assessment of nonspecific binding among different imager and docking strands pairs

To assess the specificity of interactions between different imager and docking strand pairs, we designed a series of orthogonal assays. In this experiment, three imaging strands conjugated with the same fluorescent dye (P1-AF647, P2-AF647, and P3-AF647) were sequentially applied for in situ exchange-PAINT imaging of three distinct docking strands (P1’-NC-10 nt, P2’-NC-10 nt, and P3’-NC-10 nt). Representative single-frame images from the orthogonal tests are shown in Figure S4a, indicating that observable binding events occurred only when the imager and docking strands were complementary; no significant signal was detected in non-complementary pairs. Furthermore, we quantified the binding events across multiple imaging frames (8000 frames) and normalized the counts against the maximum value within each imager strand group. As illustrated in Fig. S4b, the number of localization events resulting from nonspecific binding in non-matched imager strand–docking strand combinations was extremely low, accounting for a negligible fraction of the total binding events. These results demonstrate that nonspecific hybridization between different imager and docking strand pairs does not compromise the accurate quantification of exosomal surface markers.


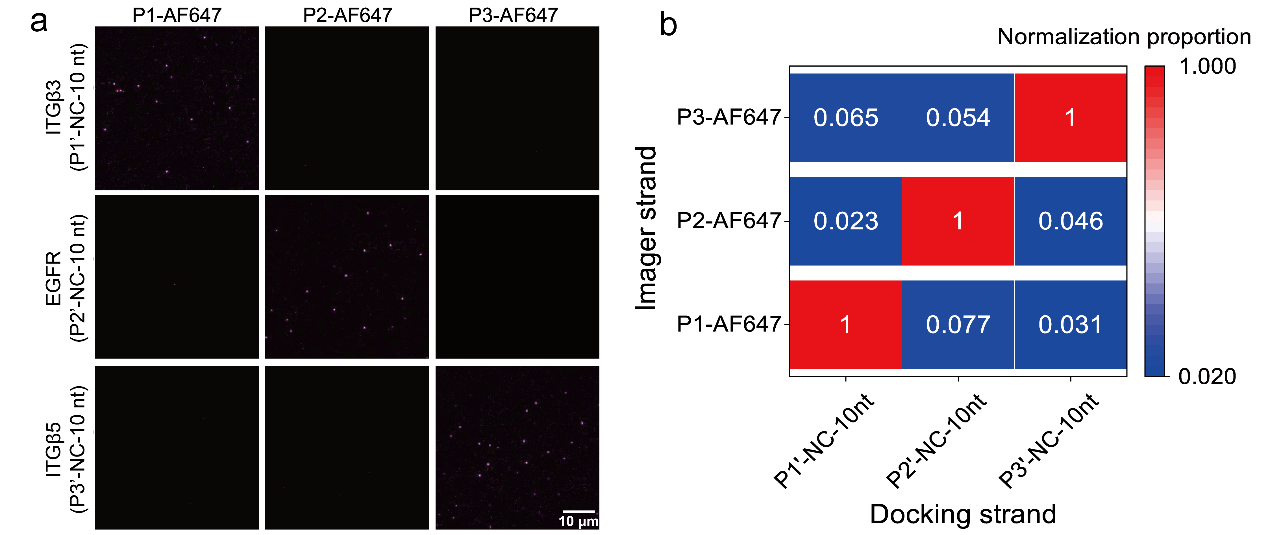


**Figure S4.** (a) Single-frame stochastic localization images from cross-validation experiments demonstrating non-specific binding events using imaging strands with identical fluorophores but different sequences for exosomal surface marker detection. (b) Heatmap of normalized binding/dissociation events across imager and docking strand combinations. Scale bars, 10 µm


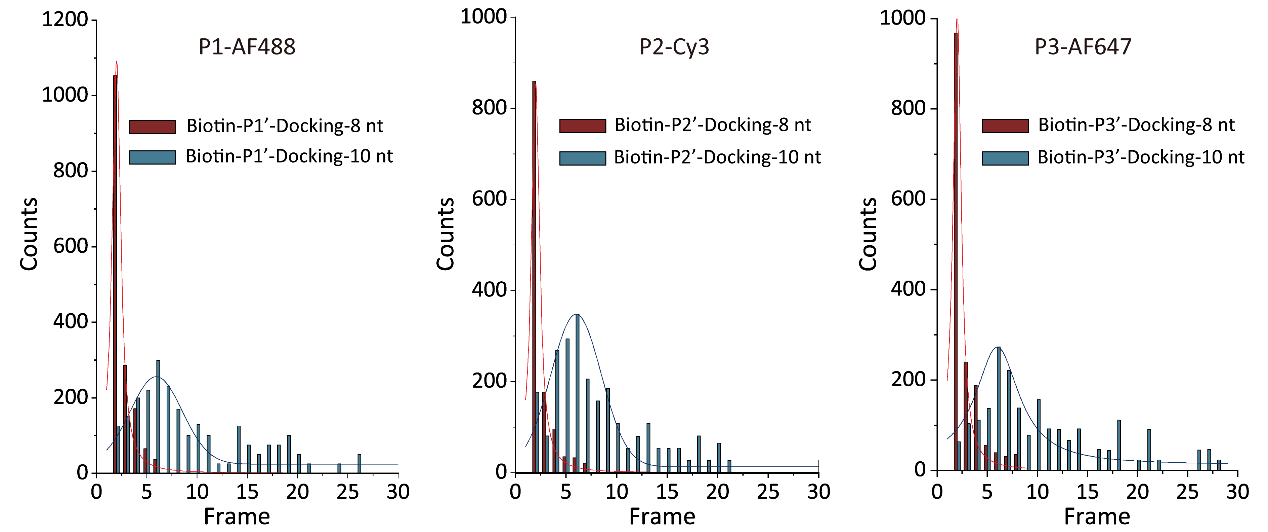


## Figure S5. Binding-time distribution of different imager-docking strand pairs. The binding-time distribution was derived from a gaussian fit.

**
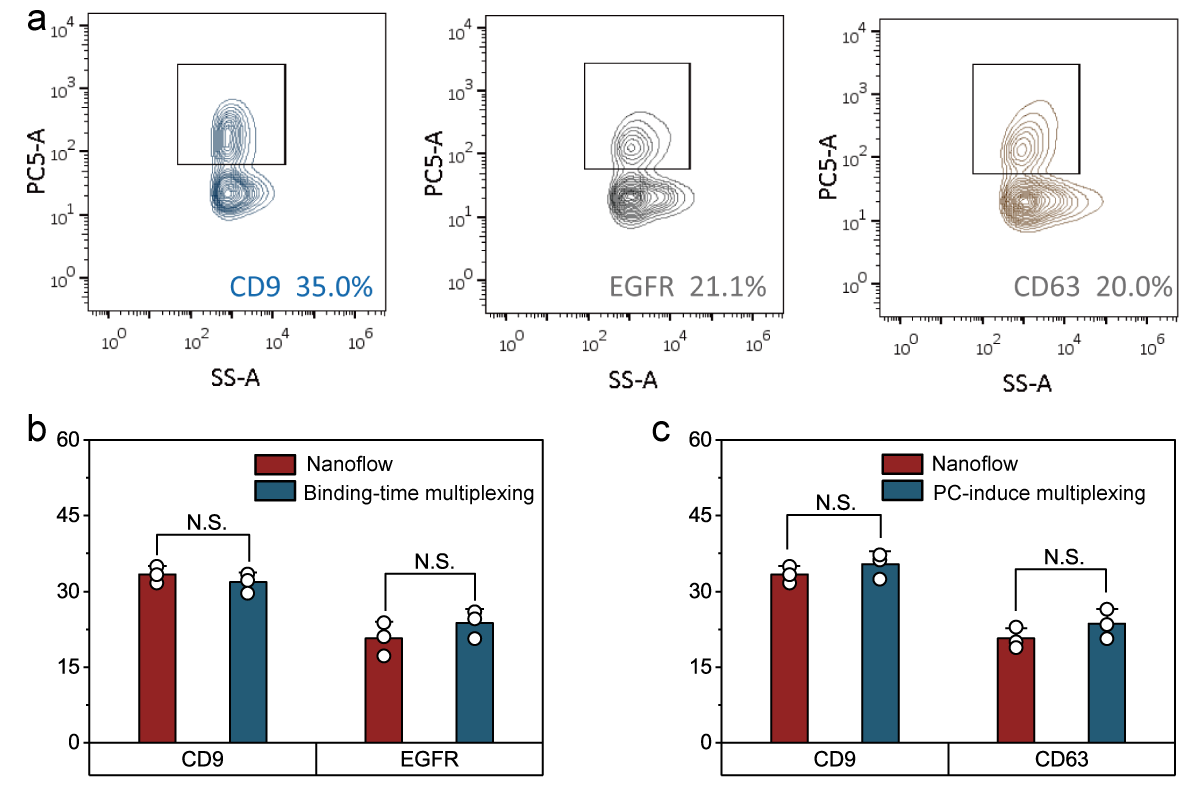
**

Figure S6. Validation of binding time and PC-induce multiplexing via nanoflow cytometry. (a) Nanoflow cytometry measurements of CD9, EGFR, and CD63 expression in A549 cell. (b) Comparative analysis of CD9 and EGFR expression on exosomes between nanoflow cytometry and binding time-dependent quantification. (c) Comparative analysis of CD9 and CD63 expression on exosomes between nanoflow cytometry and PC-induce quantification. Data are from three independent experiments and are presented as mean ± SD. Significance was determined by Tukey's test: *p < 0.05, **p < 0.01, ***p < 0.001, ****p < 0.0001; N.S., not significant.


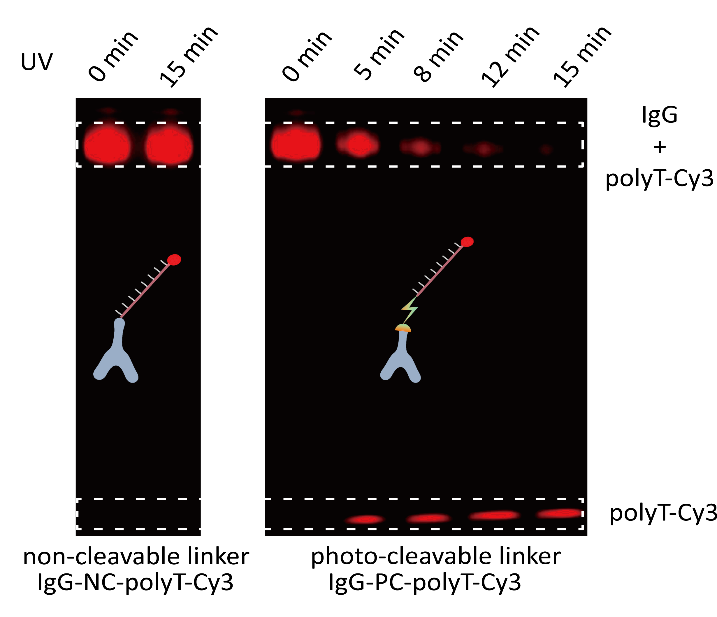


## Figure S7. SDS-PAGE electrophoretograms of goat anti-rabbit IgG-DNA-Cy3 conjugates with photo-cleavable/non-cleavable linkers under different UV irradiation time.


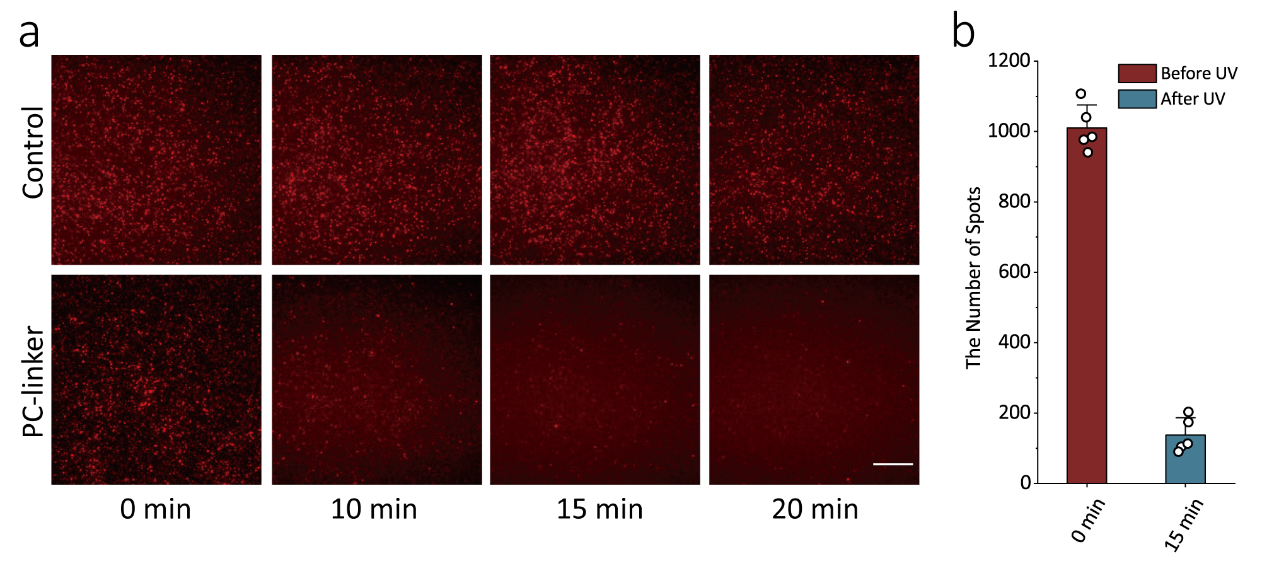
Figure S8. Assessment of photocleavage efficiency in control and PC groups. (a) Representative fluorescence microscopy images showing the distribution of fluorescence spots in the control and PC groups following UV light exposure for different durations. (b) Statistical analysis of fluorescence spot counts in the PC group before and after UV-induced photocleavage. Scale bars, 10 µm (Experimental conditions: The SA-coated substrate was incubated with 40 μL of 5 μg/mL biotinylated rabbit IgG at room temperature for 15 min. For the control group, 40 μL of 2 μg/mL goat anti-rabbit IgG-NC-polyT-Cy3 was added, while the PC-linker group received 40 μL of 2 μg/mL goat anti-rabbit IgG-PC-polyT-Cy3. The samples were exposed to UV irradiation and imaged by TIRF microscopy at designated time points. (excitation wavelength: 561 nm, exposure time: 100 ms)). Data are from five independent experiments and are presented as mean ± SD. Scale bars, 10 µm

## ****Validation of Control Group for Photo-Cleavage Response Multiplexing****

To further evaluate the accuracy of photo-cleavage-mediated multiplexed biomarker detection, we designed two control experiments. In Control group 1, CD9 and CD63 antibodies were conjugated to identical DNA docking strands via non-cleavable linkers. Upon UV irradiation of A549 cell-derived exosomes, quantitative analysis revealed consistent fluorescent spot counts in the field of view with maintained high co-localization efficiency (Figure S9), demonstrating that non-cleavable linkers remain unaffected by UV exposure. In contrast, Control group 2 employed photo-cleavable linkers to conjugate CD9 and CD63 antibodies to the same DNA docking strands. Following UV irradiation, we observed a significant reduction in localization points, confirming near-complete cleavage of all photo-cleavable linkers. These results collectively validate the feasibility of photo-cleavage-based strategies for multiplexed biomolecular detection.


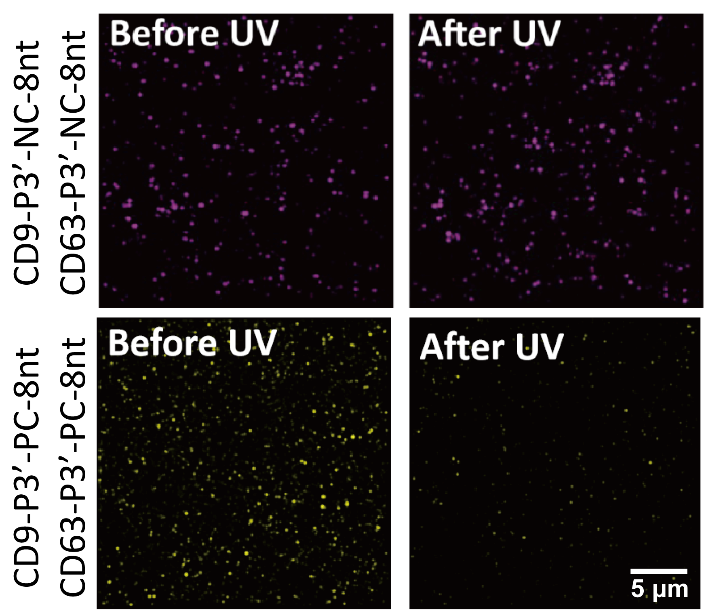


Figure S9. Representative fluorescence images of CD63 and CD9 in the control group before and after cleavage. Control group1: Both CD9 and CD63 were conjugated to the docking strand via non-cleavable linkers. Control group2: Both CD9 and CD63 were conjugated to the docking strand via photo-cleavable linkers. Scale bars, 5 µm

## ****Cross-validation of consistency among different multiplexing modes****

To validate the consistency of exosomal biomarkers quantified by spectral, binding-time, and PC-induced multiplexing, we established an orthogonal assay to detect integrin αv (P3’-NC-8 nt) and integrin β3 (P1’-PC-10 nt) on HT29-derived exosomes.

First, the sample chamber was incubated with a mixture of imager cocktail (P1-AF488, P2-Cy3, and P3-AF647). Sequential DNA-PAINT imaging was then performed using 488 nm and 640 nm lasers to quantify the expression levels of integrin αv and integrin β3 via spectral multiplexing. Subsequently, the imager cocktail was replaced with an equimolar mixture of P1-AF647, P2-AF647, and P3-AF647 for kinetic imaging under identical conditions. The resulting DNA-PAINT movies were analyzed to determine the expression levels of both biomarkers by counting binding events with different binding-times. Following this, the sample was exposed to UV light for 15 minutes to cleave the docking strand specific to integrin β3 (P1’-PC-10 nt), after which another round of imaging was conducted. The expression ratios of the two markers were calculated by counting blinking events before and after photocleavage (without differentiating binding time). As summarized in Figure S10, the expression levels of the two integrin subunits quantified by the different multiplexing strategies showed no significant differences, demonstrating strong consistency and high accuracy across the distinct multiplexing modalities.


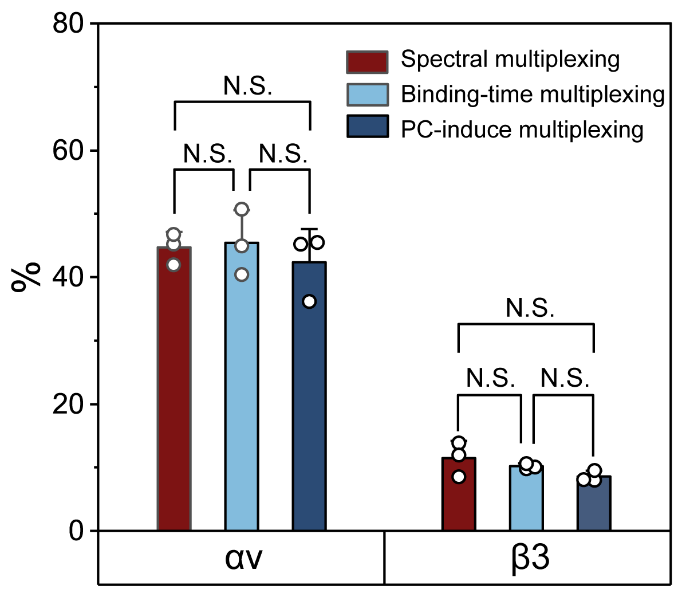


**Figure S10.** Expression levels of integrin αv and β3 determined by orthogonal assays using different multiplexing modes. Data are from three independent experiments and are presented as mean ± SD. Significance was determined by Tukey's test: *p < 0.05, **p < 0.01, ***p < 0.001, ****p < 0.0001; N.S., not significant.


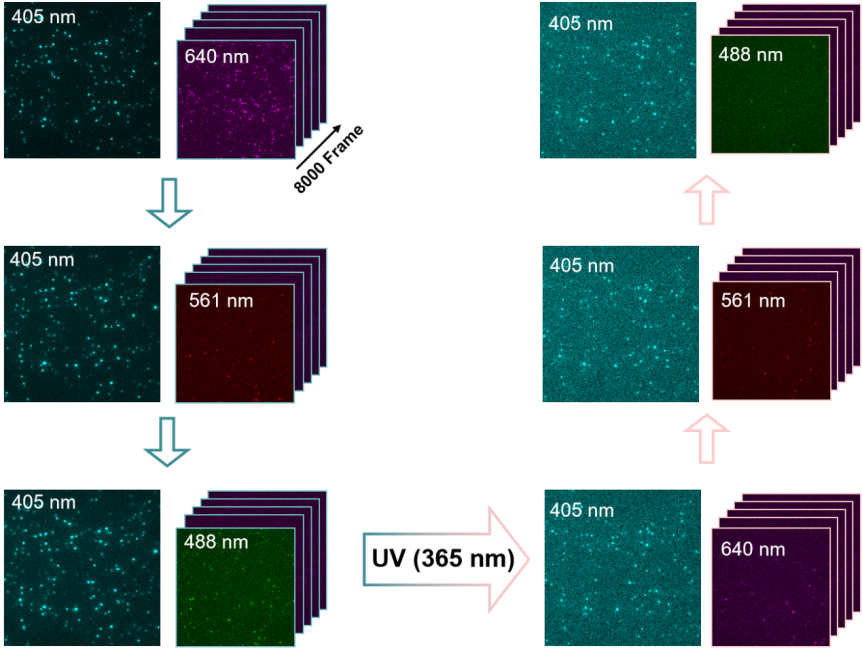


## Figure S11. Experimental workflow for single-round, wash-free multiplex detection of 12 targets

## ****Antibody Specificity Validation****

Integrin αv antibody, Integrin α6 antibody, Integrin β1 antibody，Integrin β3 antibody，Integrin β4 antibody，Integrin β5 antibody，Integrin β6 antibody and Integrin β8 antibody were employed in this study. To ensure the accuracy of exosome quantification, each antibody was applied to detect integrins on the surface of HT29 cells, thereby evaluating potential cross-reactivity or non-specific binding. Of note, the ITGβ3 antibody is not recommended for western blot applications. Therefore, it is not shown in Figure S12. For the other integrins, the observed molecular weights of the bands corresponded to the expected sizes of the target integrins and matched the positions indicated by the manufacturer. These results collectively confirm the high specificity of the antibodies used in this study.


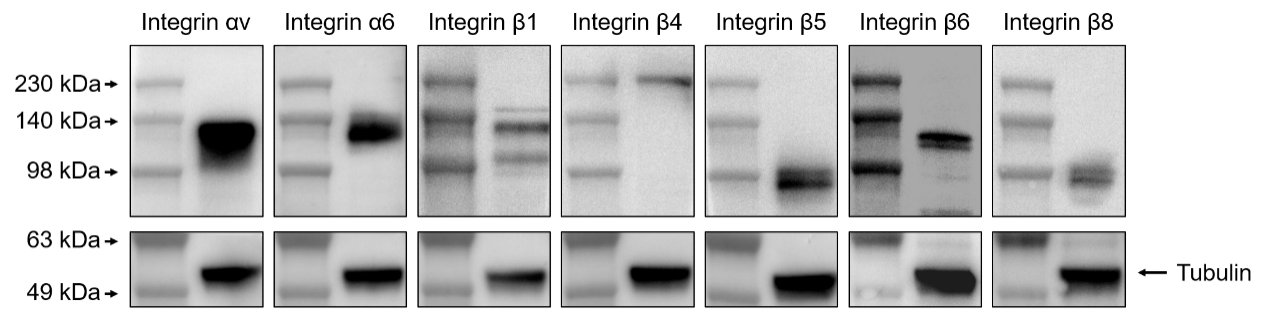


**Figure S12.** Detection of integrin expression on the surface of HT-29 cells by Western blot**.**

## Comparison of copy number obtained from 12-plex DNA-PAINT and single-target DNA-PAINT

To validate the accuracy of the copy number quantification using 12-plex DNA-PAINT, one representative marker per imaging channel (α6, CD81, and β1) was analyzed. As shown in Figure S13, the distribution of marker copy numbers obtained from 12-plex DNA-PAINT is closely aligned with that derived from individual marker analysis. This result demonstrates that the combined use of multiple multiplexing modes does not affect the accuracy of exosomal marker copy number quantification.


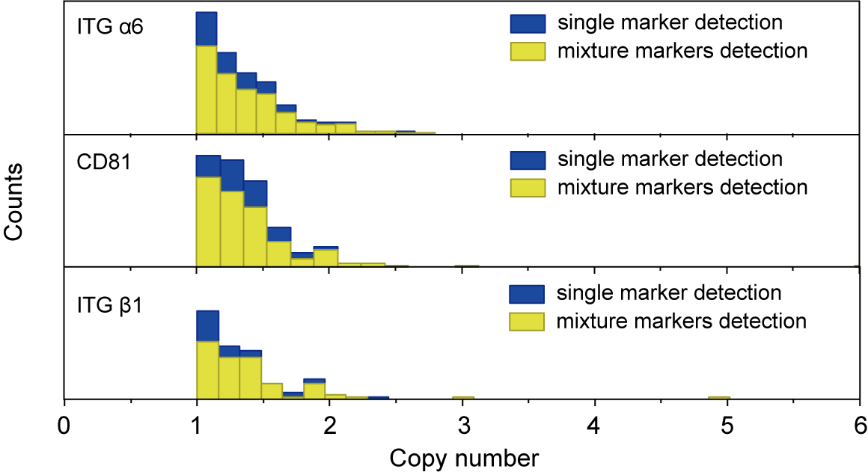


**Figure S13.** Comparison of protein copy number obtained from 12-plex DNA-PAINT and single-target DNA-PAINT.


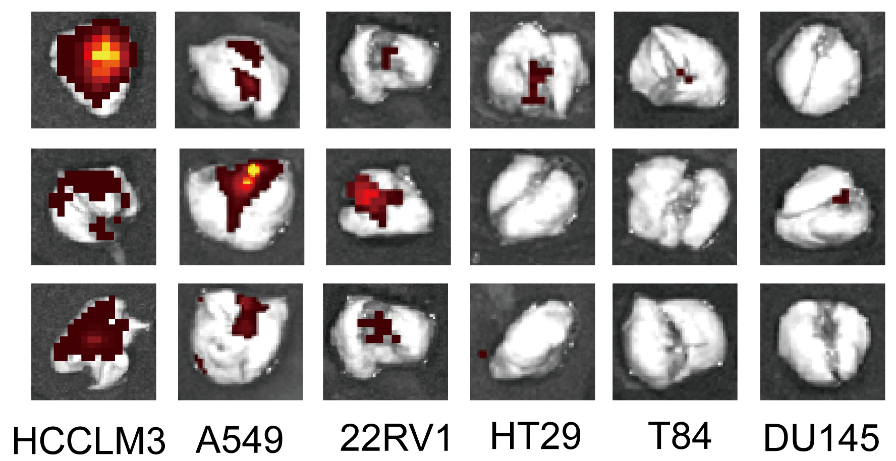


**Figure S14.** Representative fluorescence images of mouse lungs acquired 48 hours after tail vein injection of different types of exosomes (n=5).

## [Assessment of exosome enrichment in the lungs of integrin α6 blockade mice](#_Toc214054181)

To further investigate the role of the integrin α6 heterodimer in exosomal lung targeting, we compared the lung- targeting extent of exosomes in healthy with ot without integrin α6 blockade. It is important to note that we could not find any α6 integrin heterodimers antibody for the blocking experiment. Therefore, we systemically administered a well-characterized α6 subunit-blocking antibody to mice prior to exosome injection. This strategy effectively blocks all α6-containing heterodimers, including α6β1 and α6β4, as well as α6 monomer. The results showed that α6 blockade significantly inhibited lung accumulation of exosomes from both A549 and 22RV1 cell lines, which contains α6 integrin heterodimers. Considering these results and the fact that exosomes with α6 monomer, like DU145 and T84 exosomes, showed no obvious lung targeting effect, we conclude that α6-containing heterodimers are essential for lung targeting*.*


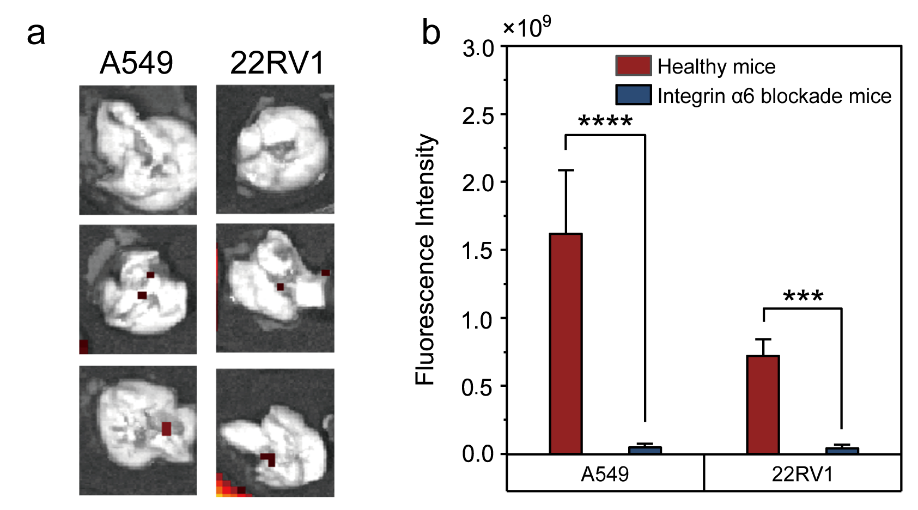


**Figure S15.** (a) Representative fluorescence images of integrin α6 mouse lungs 48 hours post-tail vein injection of A549 and 22RV1 exosomes. (b) Mean fluorescence intensity of exosomes derived from lung-tropic exosomes in mouse lungs, n= 5 technical replicates. Significance was determined by Tukey's test: *p < 0.05, **p < 0.01, ***p < 0.001, ****p < 0.0001; N.S., not significant.

## Assessment of exosome enrichment in the lungs of tumor-bearing mice

The lung targeting efficiency of A549 and HT29 exosomes was compared between A549 subcutaneous tumor-bearing mice and healthy mice. The results demonstrated that the lung enrichment of both A549- and HT29-derived exosomes showed no significant difference between the tumor-bearing and healthy mice (Figure S16). This finding provides in vivo evidence that the systemic changes induced by a subcutaneous tumor burden did not substantially alter the inherent lung-targeting capacity governed by the exosomes' surface molecular signatures in our experimental system. These results further reinforce the robustness of our central conclusion, highlighting that the exosomal integrin profile serves as a primary determinant of organotropism that operates consistently even within the complex physiological context of a tumor-bearing host


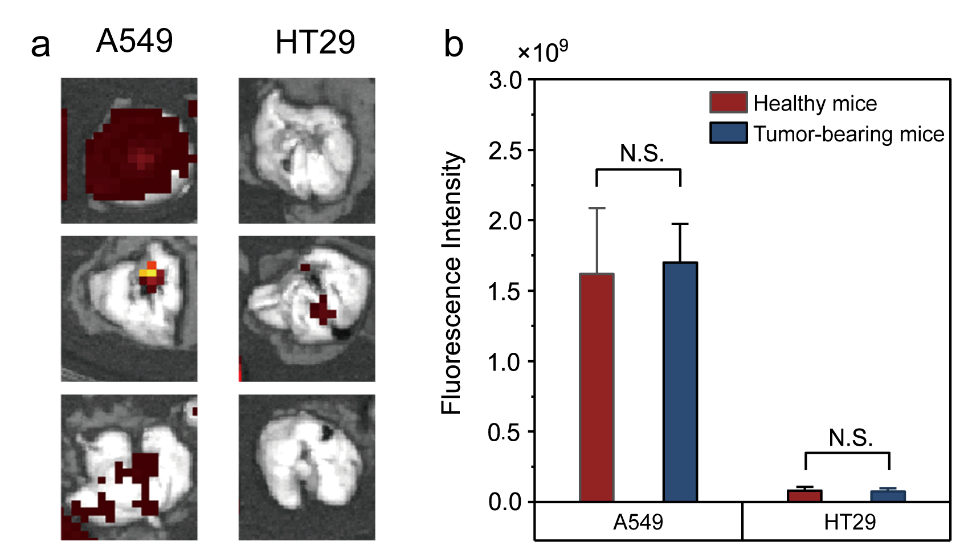


**Figure S16.** (a) Representative fluorescence images of tumor-bearing mouse lungs 48 hours post-tail vein injection of A549 and HT29 exosomes. (b) Mean fluorescence intensity of exosomes derived from lung-tropic exosomes in mouse lungs, n= 5 technical replicates. Significance was determined by Tukey's test: *p < 0.05, **p < 0.01, ***p < 0.001, ****p < 0.0001; N.S., not significant.

## Table S1. Comparison of performances between this work and other representative single-exosome analysis methods.

| Method (corporation) | Multiplexing capability | Resolution  (nm) | Sensitivity | Copy number calculation |
| --- | --- | --- | --- | --- |
| Nanoimager (ONI) | 4 | 20 | Single biomarker | × |
| Exoview (Nanoview) | 4 | 230 | Single vesicle | × |
| NanoFCM | 2 | NA | Single vesicle | × |
| This work | 12 | 230 | Single biomarker | √ |

## Table S2. Influx rates (ξ) of different imager strands and docking strands calculated via single-stranded DNA

| **Laser (nm)** | **Complementary based numbers (nt)** | **Influx rate ξ (s^-1^)** |
| --- | --- | --- |
| **488** | **8** | **0.00375** |
| **488** | **10** | **0.00435** |
| **561** | **8** | **0.00388** |
| **561** | **10** | **0.00426** |
| **640** | **8** | **0.00374** |
| **640** | **10** | **0.0047** |

## ****Table S3.** Imager strands and docking strands**


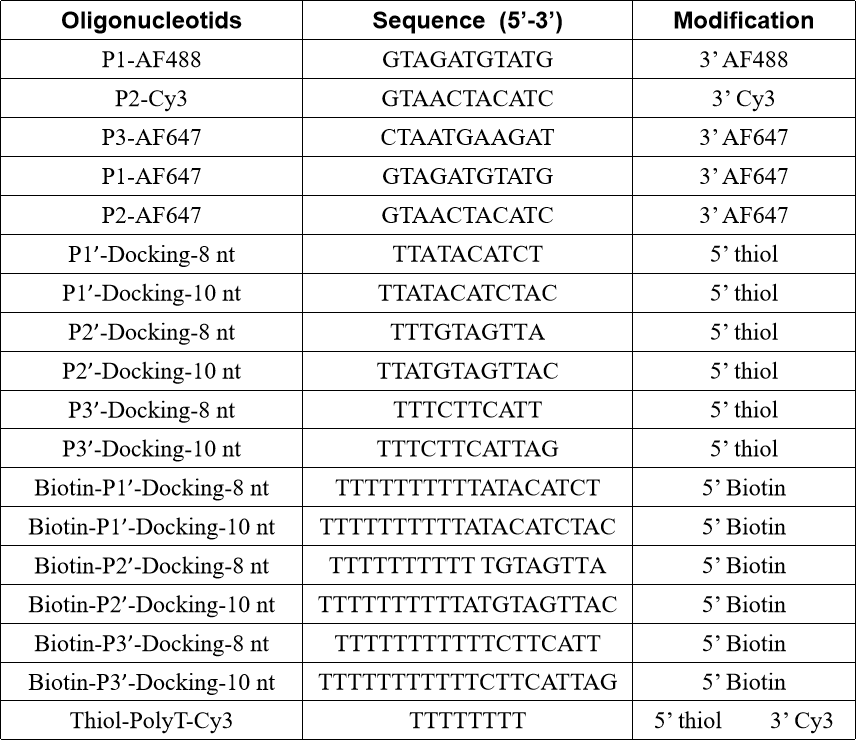


## ****Table S4.** DNA sequences for the synthesis of DNA tetrahedra**

**
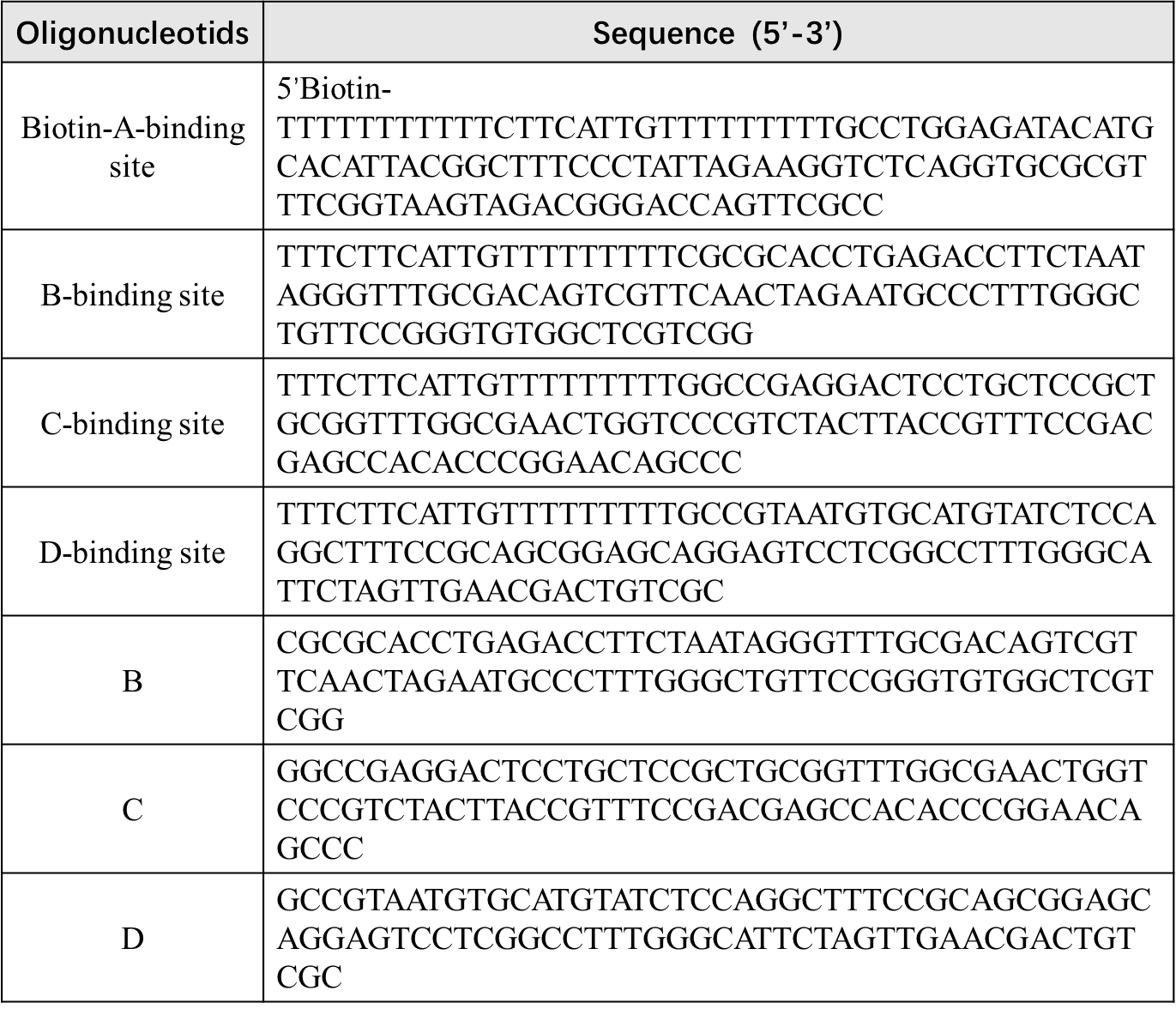
**

# ****References****

[1] S. D. Chandradoss, A. C. Haagsma, Y. K. Lee, J. H. Hwang, J. M. Nam, C. Joo, “Surface passivation for single-molecule protein studies” *J. Vis. Exp.* **2014**.,86,1-8.

[2] J. Schnitzbauer, M. T. Strauss, T. Schlichthaerle, F. Schueder, R. Jungmann, “Super-resolution microscopy with DNA-PAINT” *Nat. Protoc.* **2017**, *12*, 1198-1228.

[3] Y. Jiang, J. Zhang, S. R. Jung, H. Chen, S. Xu, D. T. Chiu, “High-Precision Mapping of Membrane Proteins on Synaptic Vesicles using Spectrally Encoded Super-Resolution Imaging” *Angew. Chem. Int. Ed. Engl.* **2023**, *62*, e202217889.

[4] M. Lin, J. Wang, G. Zhou, J. Wang, N. Wu, J. Lu, J. Gao, X. Chen, J. Shi, X. Zuo, C. Fan, “Programmable engineering of a biosensing interface with tetrahedral DNA nanostructures for ultrasensitive DNA detection” *Angew. Chem. Int. Ed. Engl.* **2015**, *54*, 2151-2155.

[5] O. K. Wade, J. B. Woehrstein, P. C. Nickels, S. Strauss, F. Stehr, J. Stein, F. Schueder, M. T. Strauss, M. Ganji, J. Schnitzbauer, H. Grabmayr, P. Yin, P. Schwille, R. Jungmann, “124-Color Super-resolution Imaging by Engineering DNA-PAINT Blinking Kinetics” *Nano. Lett.* **2019**, *19*, 2641-2646.

[6] L. V. D. Maaten, G. Hinton, “Visualizing Data using t-SNE”, *J. Mach. Learn. Res,* **2008,** 9, 2579-2605.
